# Supplementary material for: Fronto-medial theta coordinates posterior maintenance of working memory content
Source: Curr Biol. 2022 May 23;32(10):2121–2129.e3. doi: 10.1016/j.cub.2022.03.045 (PMC9616802; doi:10.1016/j.cub.2022.03.045)
Supplement: Document S1. Figures S1–S5 and Table S1 [file mmc1.pdf]

Current Biology, Volume *32*

Supplemental Information

Fronto-medial theta coordinates posterior maintenance of working memory content

Oliver Ratcliffe, Kimron Shapiro, and Bernhard P. Staresina

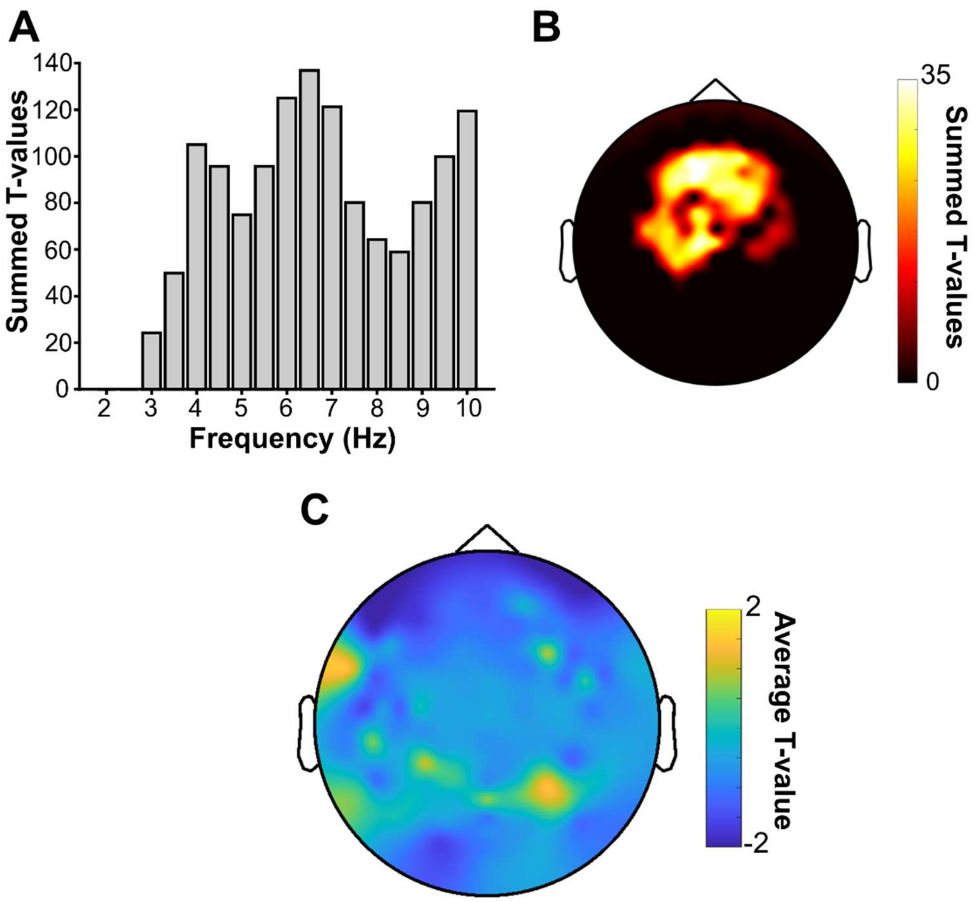

Figure S1. WM-induced fronto-medial theta (FMT) power in the delay period of the 1-back task (with an alternative baseline comparison) and in the delay period of the DMS task. Related to Figure 2. Cluster-corrected comparison of oscillatory power in the delay period of the 1-back task relative to an inter-block baseline period, revealing a significant increase in theta power (4-8 Hz; **A**, summed across significant channels) at fronto-medial channels (**B**, summed across significant frequencies from 4-8 Hz). **C.** Theta power in the delay period of the DMS task relative to the pre-stimulus baseline. No significant clusters emerged after correcting for multiple comparisons. The uncorrected topography (averaged over frequencies in the theta range) indicated that power was primarily increased over posterior channels, albeit without reaching statistical significance.

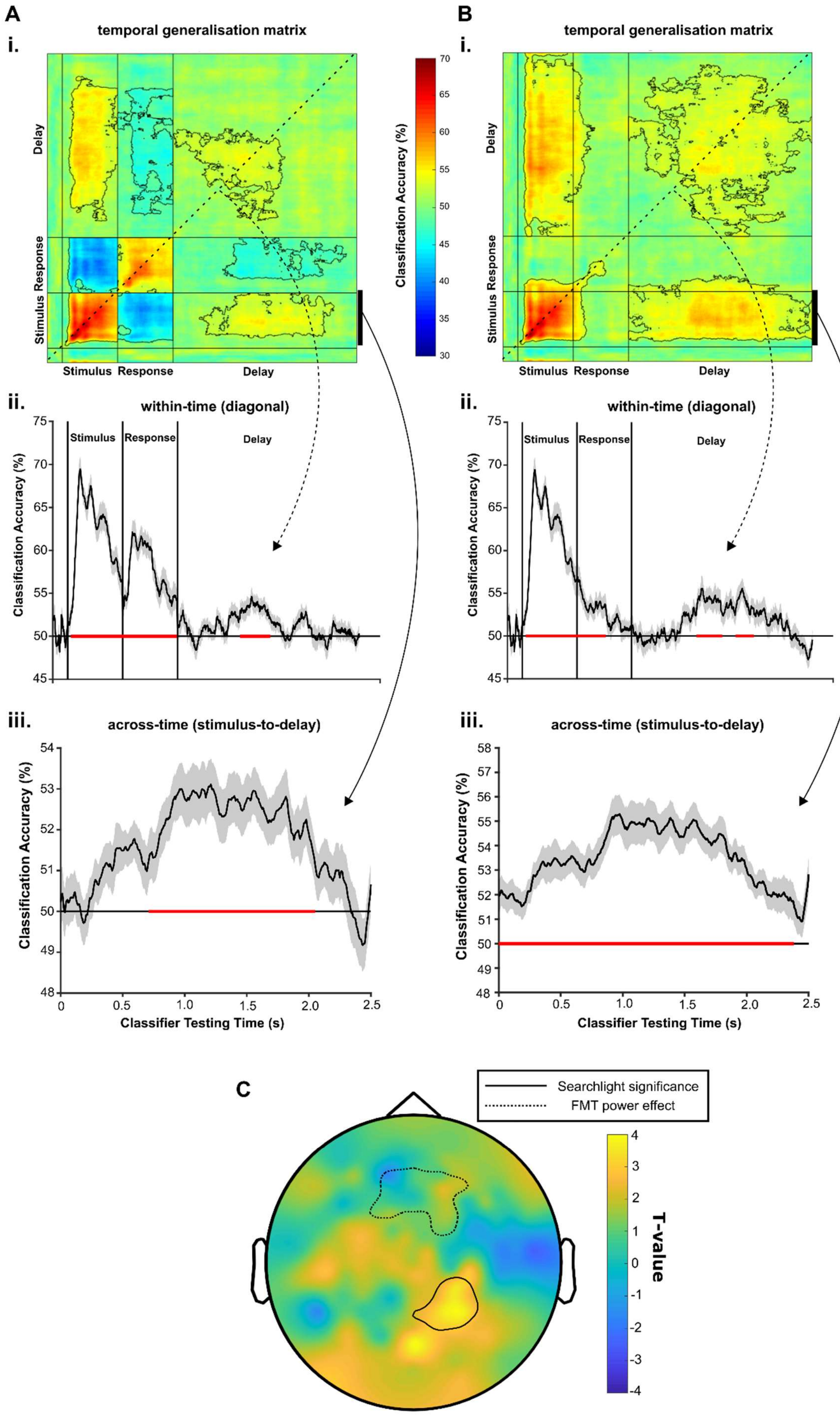

A
B
C
i.
temporal generalisation matrix
Delay
Stimulus Response
Stimulus Response Delay
Classification Accuracy (%)
70
65
60
55
50
45
40
35
30
ii.
within-time (diagonal)
Stimulus
Response
Delay
iii.
across-time (stimulus-to-delay)
Classifier Testing Time (s)
Searchlight significance
FMT power effect
T-value

Figure S2. Multivariate decoding across the full trial. Related to Figure 3. A.i. Classifiers trained and tested on every time point of each period of 1-back trials revealed generalisation between the stimulus and delay periods. Black outlines indicate cluster-corrected significance ($p < 0.05$). **A.ii.** Training and testing on the same time periods reveals significant decoding above chance in the stimulus, response, and delay periods. Note that these data reflect the diagonal of A.i. **A.iii.** Classifier accuracy was averaged across the training dimension during the period when the stimulus was on the screen (0-750 ms), revealing significant generalisation (as shown by above-chance accuracy) during the delay period. **B.** same as A., but conducting baseline correction using only the pre-stimulus period. **C.** Un-thresholded searchlight decoding topography. Topography of testing searchlight decoding accuracy against chance (cluster-corrected t-test); as in Figure 3B but plotting all channels (rather than only the significant cluster). Searchlight decoding was performed by using taking each channel and its immediate neighbours and decoding during the previously defined temporal window of interest (860-1275 ms). The value for each channel indicates the t-value (comparing the decoding performance relative to chance) when this channel was used as the centre of the decoding searchlight. The solid outline indicates cluster-corrected significance. To contrast, the dotted black line indicates channels showing the WM-induced FMT power difference (as in Figure 2B).

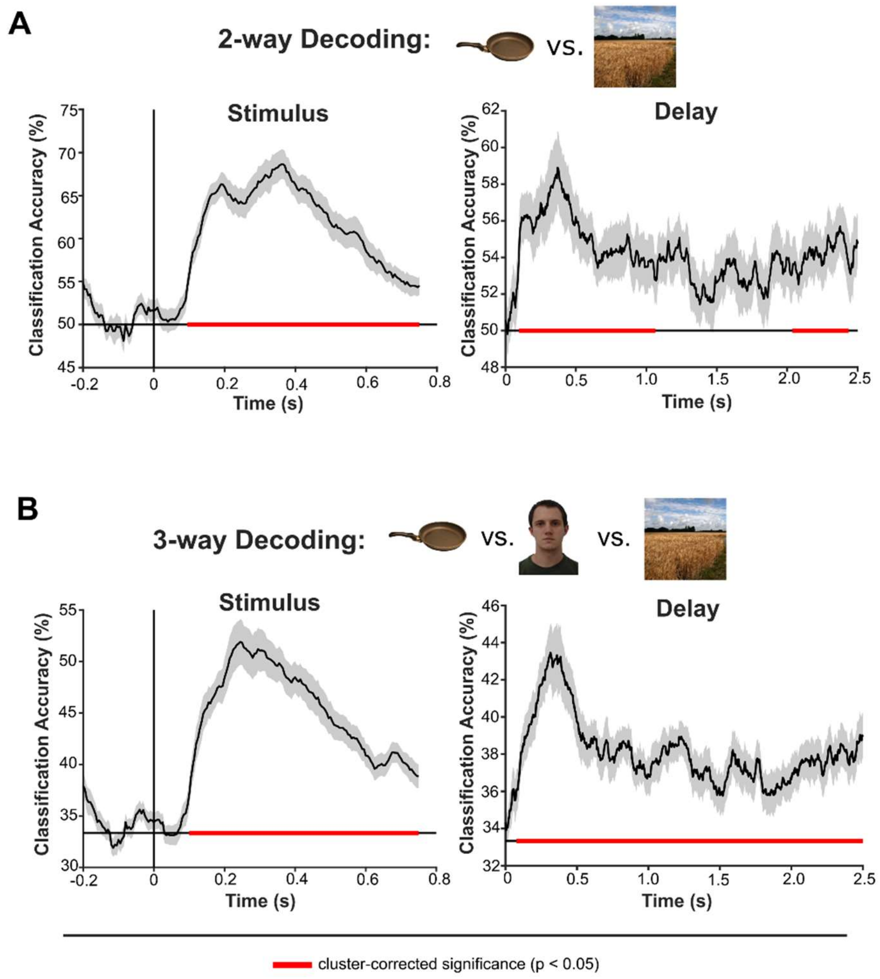

Figure S3. Decoding category of maintained content in the DMS task. Related to STAR★Methods. In order to test generalisability of 1-back maintenance decoding of WM representations to a similar task, we trained and tested classifiers on the stimulus and delay periods of the DMS task (main text Figure 1).

A. Binary decoding accuracy (classifying between object and scene trials) was significantly above chance during the stimulus and delay period intervals, as well as when averaging accuracy over the full time in each period [stimulus, $t_{(27)} = 11.46$, $p < 0.001$, Cohen's $d = 2.17$; delay, $t_{(27)} = 5.07$, $p < 0.001$, Cohen's $d = 0.96$]. **B.** Decoding between the three categories present in the DMS task (object/scene/face) was also above-chance during the stimulus and delay periods both when examining accuracy across-time and when averaging over the time dimension [$t_{(27)} = 10.87$, $p < 0.001$, Cohen's $d = 2.05$; delay, $t_{(27)} = 6.97$, $p < 0.001$, Cohen's $d = 1.32$].

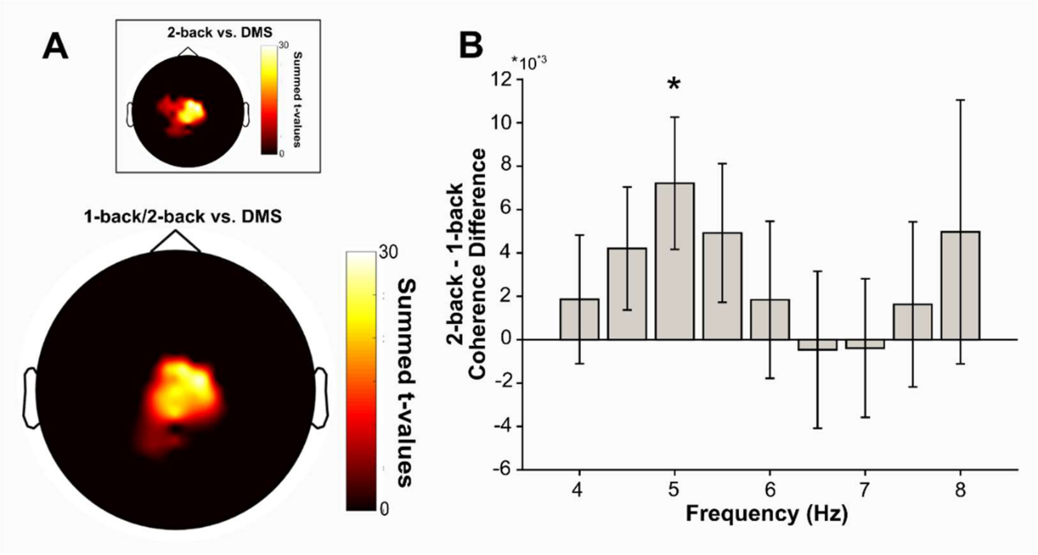

Figure S4. Theta coherence with increasing WM load. Related to STAR★Methods. A. Cluster-corrected comparison revealed an extended cluster showing increased coherence during the delay period of both the 2-back and 1-back tasks relative to the DMS task. T-values were summed across significant frequencies for each comparison and then averaged over the 1-back and 2-back tasks. **Inset.** Cluster showing significantly increased coherence in the delay period of the 2-back task relative to the DMS task. **B.** Averaging across the full delay period and significant channels, subtracting coherence values in the 1-back task from the 2-back task revealed a shift to the lower end of the theta band. This was evidenced by coherence at 5 Hz being significantly greater in the 2-back task relative to the 1-back task. Error bars indicate the standard error of the mean and the asterisk indicates statistical significance at alpha = 0.05.

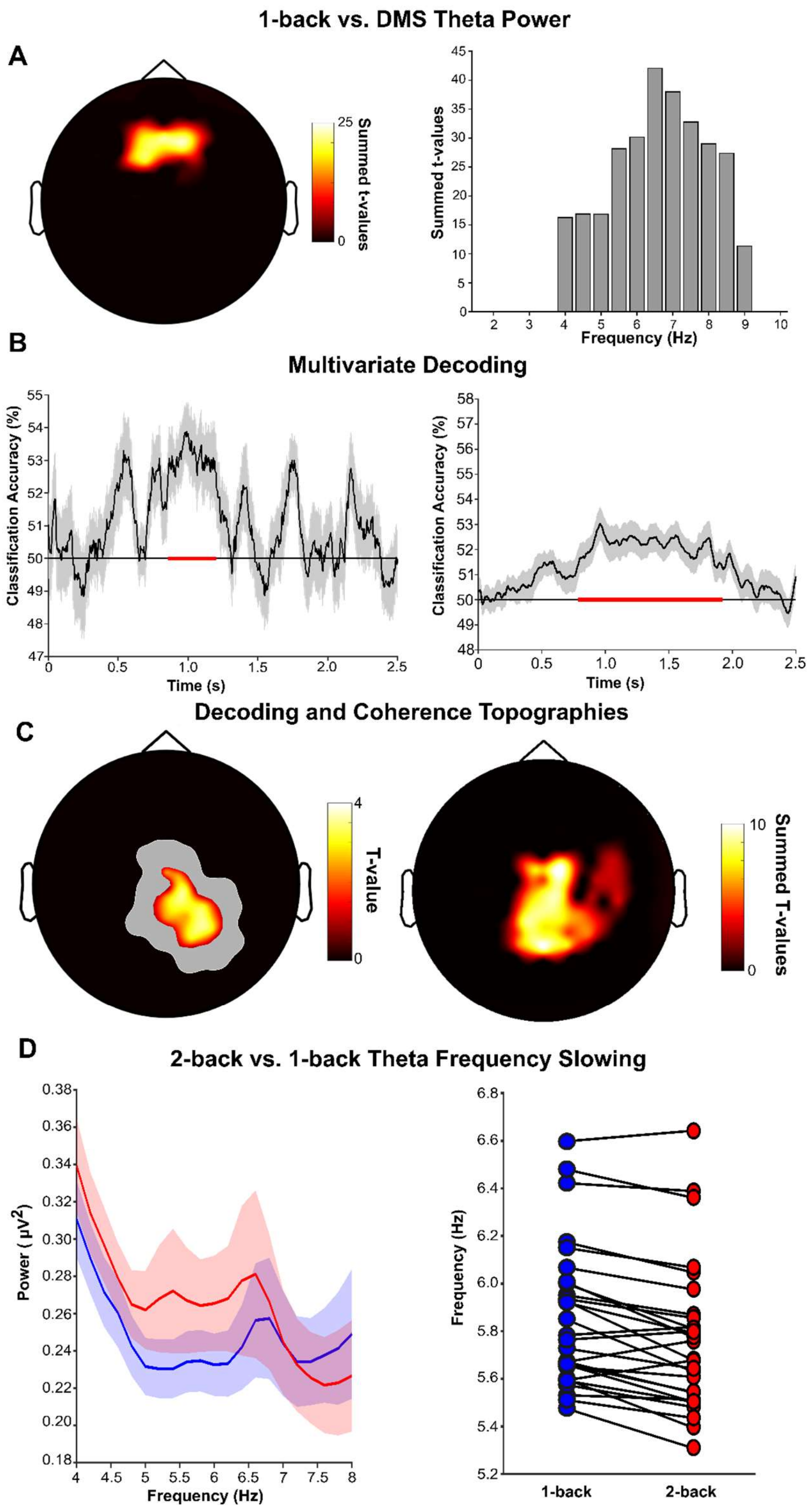

Figure S5. Analyses replicated using only correct trials. Related to STAR★Methods. A. FMT power effect comparing the delay period of the 1-back task to the same period during the DMS task. **B.** Accuracy of a classifier decoding the category of maintained content was significantly above chance during the delay period, both when training the classifier on that same period of the task **(left)** and when training on the stimulus presentation period of the task **(right)**. **C.** A decoding searchlight revealed that channels driving the significant decoding of stimulus representations showed substantial overlap with channels showing coherence between FMT channels in A. **D.** As when analysing all trials, the frequency of the FMT power effect significantly decreased in frequency (slowed) with the addition of a second to-be-remembered item during the delay period of correct trials

Comparison	t-value	p-value	Cohen's d	Cluster-corrected time (ms)
**Stimulus-to-delay generalisation**	4.40	<0.001*	0.83	710-2050
**Decoding with channels showing coherence with FMT activity**	2.46	0.02*	0.46	700-970

Table S1. Decoding across the full delay period. Related to STAR★Methods. Table displaying t-values, p values, and effect sizes averaging classifier accuracies (columns 2-4) and the time course of cluster-corrected periods of above-chance accuracy (column 5) across the full delay period (t-tests against chance). Asterisk indicates statistical significance ($p < 0.05$).
