# Supplementary material for: Fronto-medial theta coordinates posterior maintenance of working memory content
Source: Curr Biol. 2022 May 23;32(10):2121–2129.e3. doi: 10.1016/j.cub.2022.03.045 (PMC9616802; doi:10.1016/j.cub.2022.03.045)
Supplement: Document S2. Article plus supplemental information [file mmc2.pdf]

Current Biology

Article

Fronto-medial theta coordinates posterior maintenance of working memory content

Highlights

- FMT power supports WM functions
- During WM performance, posterior/parietal regions are coupled with FMT
- Multivariate decoding of WM content is mediated by these same posterior channels
- Frontal theta frequency slows with WM load supporting phase-coding models

Authors

Oliver Ratcliffe, Kimron Shapiro, Bernhard P. Staresina

Correspondence

bernhard.staresina@psy.ox.ac.uk

In brief

Ratcliffe et al. show working memory (WM) demands increase theta power (4–8 Hz) in fronto-medial cortex. WM content, in turn, is decodable from posterior areas showing functional coupling with frontal cortex. Finally, holding an additional item in WM significantly slows the frontal theta rhythm, supporting phase-coding accounts of WM function.

Ratcliffe et al., 2022, Current Biology *32*, 2121–2129
May 23, 2022 © 2022 The Author(s). Published by Elsevier Inc.
https://doi.org/10.1016/j.cub.2022.03.045

CellPress

Current Biology

CellPress
OPEN ACCESS

Article

Fronto-medial theta coordinates posterior maintenance of working memory content

Oliver Ratcliffe,[1] Kimron Shapiro,[1] and Bernhard P. Staresina[2,3,4,*]
[1]School of Psychology, University of Birmingham, Edgbaston, Birmingham B15 2TT, UK
[2]Department of Experimental Psychology, University of Oxford, Oxford, UK
[3]Oxford Centre for Human Brain Activity, Wellcome Centre for Integrative Neuroimaging, Department of Psychiatry, University of Oxford, Oxford, UK
[4]Lead contact
*Correspondence: bernhard.staresina@psy.ox.ac.uk
https://doi.org/10.1016/j.cub.2022.03.045

SUMMARY

How does the human brain manage multiple bits of information to guide goal-directed behavior? Successful working memory (WM) functioning has consistently been linked to oscillatory power in the theta frequency band (4–8 Hz) over fronto-medial cortex (fronto-medial theta [FMT]). Specifically, FMT is thought to reflect the mechanism of an executive sub-system that coordinates maintenance of memory contents in posterior regions. However, direct evidence for the role of FMT in controlling specific WM content is lacking. Here, we collected high-density electroencephalography (EEG) data while participants engaged in WM-dependent tasks and then used multivariate decoding methods to examine WM content during the maintenance period. Engagement of WM was accompanied by a focal increase in FMT. Importantly, decoding of WM content was driven by posterior sites, which, in turn, showed increased functional theta coupling with fronto-medial channels. Finally, we observed a significant slowing of FMT frequency with increasing WM load, consistent with the hypothesized broadening of a theta "duty cycle" to accommodate additional WM items. Together, these findings demonstrate that frontal theta orchestrates posterior maintenance of WM content. Moreover, the observed frequency slowing elucidates the function of FMT oscillations by specifically supporting phase-coding accounts of WM.

INTRODUCTION

Working memory (WM) is the ability to retain and manipulate information over short delays.[7] It is thought to rely on at least two functionally distinct sub-systems.[8] The first is an executive system, which directs cognitive resources and oversees the prioritization and readout of representations. This system interacts with the representational system, which directly holds task-relevant informational content. These two systems rely on disparate brain regions, in particular frontal and parietal areas.[9–12] Importantly, WM requires functional interactions between these systems. A prime mechanism to facilitate inter-regional communication in service of WM is oscillatory activity in the theta range (4–8 Hz).[13,14]

Specifically, according to an influential computational framework,[15–17] theta oscillations provide gated processing windows in which neural representations of target information ("WM items") become active. Consistent with this model, non-human primate work has demonstrated that neurons coding for item-related information preferentially fire during particular theta phases.[18] Similarly, a recent study using intracranial recordings in humans has shown nesting of stimulus-related gamma activity (>30 Hz) in specific theta phases.[19] Theta oscillations may thus constitute the mechanism governing goal-directed interactions between frontal executive and parietal representational sub-systems.[20]

WM tasks consistently induce theta power increases at fronto-medial sites (fronto-medial theta, FMT).[21–23] FMT is generally considered to originate locally in the medial prefrontal and anterior cingulate cortices,[24] based on source-modeling.[25–27] FMT has been linked to behavioral WM performance,[28,29] as well as to individual WM capacity.[30] Consistent with this role for FMT, transcranial magnetic stimulation (TMS) in the theta range improved WM performance.[31] Importantly, non-invasive electrophysiological studies have detected fronto-parietal coupling via theta oscillations during WM tasks.[32–34] Additionally, experimental induction of fronto-parietal theta coupling via transcranial alternating current stimulation (tACS) increased WM performance.[35,36] Finally, electroencephalography (EEG) latency analyses between frontal and parietal regions suggest that the frontal cortex is the driver of these inter-regional interactions.[37]

These findings suggest the intriguing scenario that WM content, represented and maintained in posterior/parietal cortex, is orchestrated by frontal control mechanisms via theta oscillations. However, at present it is unclear (1) whether parietal cortex represents individual items held in WM and (2) whether those parietal WM memoranda are, in turn, coordinated by FMT. Finally, it is unclear how theta rhythms orchestrate the storage of multiple items, e.g., maintaining two instead of one item in WM. On the one hand, this additional demand may be accommodated by

© 2022 The Author(s). Published by Elsevier Inc.
This is an open access article under the CC BY license (http://creativecommons.org/licenses/by/4.0/).

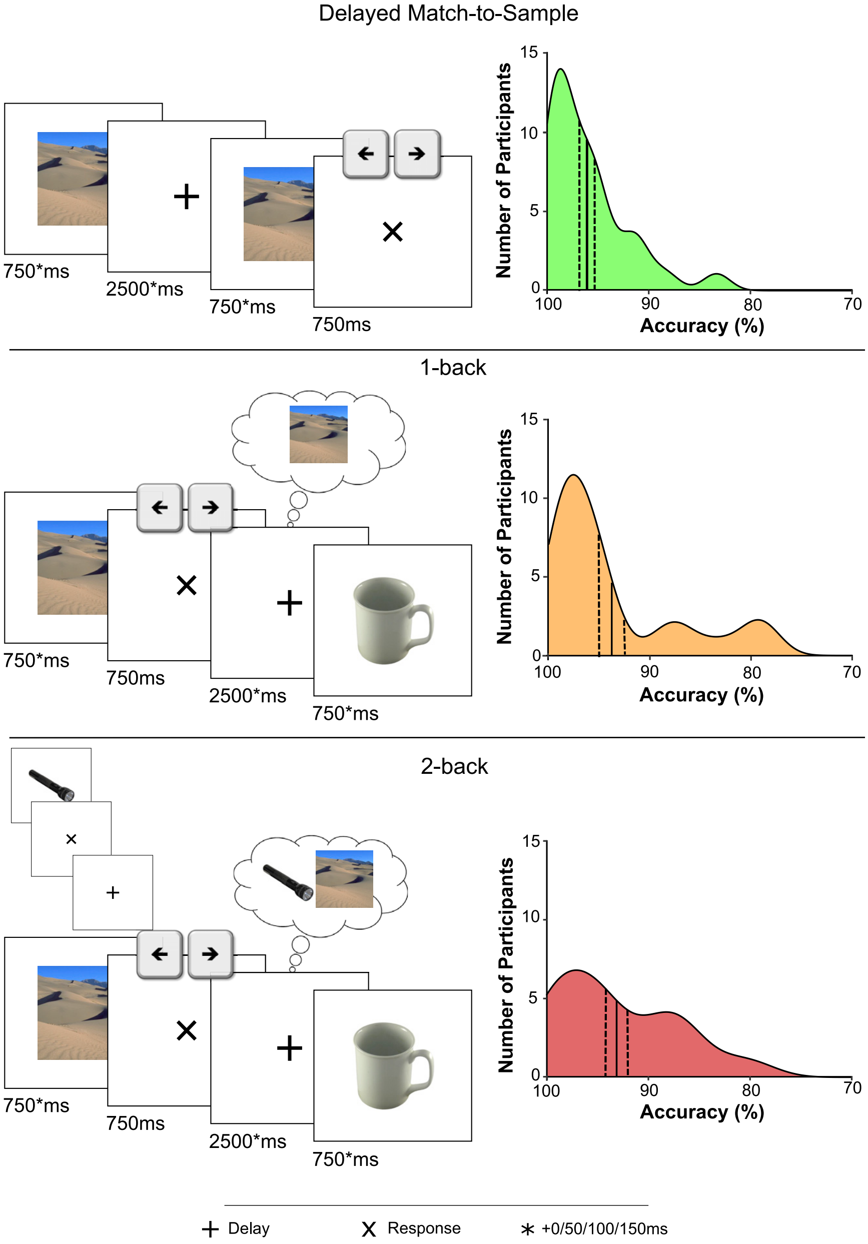

Figure 1. Experimental paradigm and behavioral results

Trial design (left) and distributions of behavioral accuracy (right) in three WM tasks performed during acquisition of EEG: a delayed-match-to-sample (DMS) task (top), a 1-back task (middle), and a 2-back task (bottom). Solid black lines indicate the mean behavioral accuracy and dashed black lines indicate the standard error of the mean across participants.

significant coherence in the theta band with the frontal channels identified previously. Finally, we found that theta frequencies slow with an additional to-be-remembered item in WM, consistent with phase-coding accounts of WM maintenance. Together, these results elucidate the role of theta rhythms for linking frontal control mechanisms with posterior content representations during WM maintenance.

RESULTS

Oscillatory mechanisms of WM maintenance

Our first goal was to confirm the role of FMT in WM maintenance. We therefore employed a 1-back task, requiring participants to encode, maintain, compare, and drop items in a continuous stream of stimuli. As a control condition, we used a delayed match-to-sample (DMS) task, which is similar in its general structure but crucially different in the level of involvement of the WM executive (Figure 1). Although both tasks require WM engagement, finer control over the same number of items is necessary in the 1-back task, where items must be selectively dropped/maintained to ensure valid comparison of the relevant stimuli. Previous work has compared a 1-back task with a variant of the DMS task (the Sternberg task) and demonstrated an increase in FMT power,[39] likely due to this greater requirement for precise control of WM representations and the need to maintain the temporal order of items to which FMT activity is responsive.[40]

Analysis of behavioral accuracy confirmed increased task difficulty for the 1-back task compared with the DMS task, despite high performance in both tasks (Figure 1; 1-back: 94% mean accuracy; SD = 6.59%; DMS: 96% mean accuracy; SD = 3.97%, paired-samples t test, $t_{(27)} = -2.40$; $p = 0.024$, Cohen's $d = 0.45$).

To examine oscillatory activity, spectral power was calculated in the delay period of both the 1-back and the DMS tasks, averaging power values across the full 2.5 s delay period. A permutation-based cluster-corrected paired-samples t test was then conducted on these channel-frequency spectra. Results

an increase in theta amplitude/power, reflecting the participation of larger neuronal assemblies.[38] Conversely, the theta-gamma framework referred to above emphasizes a role of oscillatory phase, which allows for separation of individual chunks of information.[15] Accordingly, increasing load by requiring maintenance of an additional stimulus might induce slowing of an individual's theta frequency—the slower the frequency, the more item-coding assemblies can fire within a given cycle.

To answer these questions, we designed a paradigm in which we systematically varied WM demand and decoded the category of a maintained visual stimulus while recording high-density EEG. We first show that frontal theta power increased with increased WM engagement. Second, individual items held in WM were decodable from the EEG signal of posterior channels. Intriguingly, the same channels that enabled decoding showed

CellPress
OPEN ACCESS

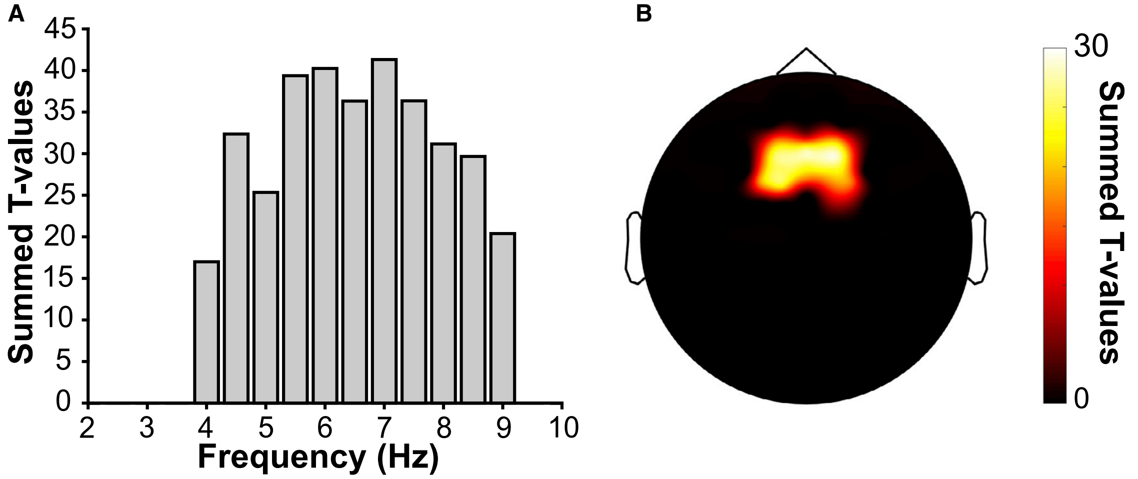

Figure 2. WM-induced fronto-medial theta (FMT) power

(A and B) Cluster-corrected comparison of oscillatory power in the delay period of the 1-back task relative to the same period in the DMS task revealed a significant increase in theta power (A) (4–8 Hz; summed across significant channels) at fronto-medial channels (B) (summed across significant frequencies from 4 to 8 Hz—note the same topography of significant channels emerged when summing across the full 4–9 Hz range). Theta power relative to the inter-block baseline period of the 1-back task and in the DMS delay period is shown in Figure S1.

revealed a significant cluster in which theta power was greater in the 1-back task as compared with the DMS task. This cluster was centered primarily around 5.5–7 Hz but spanned the entire theta frequency band (4–8 Hz; Figure 2A). Collapsing across the 4–8 Hz frequency range illustrates that this effect is driven by fronto-medial channels (Figure 2B), consistent with previous work identifying fronto-medial sources of WM-related theta oscillations.[41,42] To determine directly the effect size of this increase, power values were first averaged across the 4–8 Hz range, across the full time period and over significant channels (identified by the cluster-based permutation effect). This recapitulated the result of the cluster test ($t_{(27)} = 3.44$, $p = 0.002$) and showed that this effect was in the moderate to large range (Cohen's $d = 0.65$).[43] Previous work has linked increased WM engagement (as reflected in reduced behavioral accuracy) to increased FMT power[28] in which individuals showing a greater load-induced accuracy difference showed greater FMT power. The authors reported that this correlation was stronger when including both correct and incorrect trials but that it was not dependent on error trial inclusion. Although the same trend was evident in the present data, this correlation was only significant when analyzing all trials ($r_{(27)} = -0.40$, $p = 0.04$). When analyzing only correct trials, this correlation went in the same direction but was not statistically significant ($r_{(27)} = -0.25$, $p = 0.20$).

To ensure that the FMT effect we observed does not hinge on the comparison with the DMS task, we contrasted the maintenance period of the 1-back task to a neutral baseline condition. Although a pre-stimulus baseline period was not possible in the 1-back task (in which information must be held between trials), a 10 second inter-block baseline period was acquired before and after each block of the n-back task (STAR Methods). When power in the delay period was compared with this inter-block baseline, a FMT cluster again emerged over frontal channels (Figures S1A and S1B), similar in topography to comparing the 1-back task with the DMS task (albeit less well circumscribed). Averaging power across the full delay period and the pre-stimulus baseline period of the DMS task, a cluster-corrected t test revealed no significant clusters in the theta range (4–8 Hz). Unthresholded comparisons showed no evidence of a significant increase at fronto-medial sites (Figure S1C).

Decoding stimulus category during WM maintenance

We next addressed the question of when and where WM content is maintained during the delay period. To this end, multivariate pattern classification was applied to the delay period of object and scene trials of the 1-back task. Specifically, the ability to decode stimulus content was assessed via linear discriminant analysis (LDA) in a k-fold cross-validation regimen using—at each time point—the raw EEG signal across channels as features (see STAR Methods section for additional details). Classifier accuracy was compared with chance (50%) and corrected across time by cluster-based permutation. As shown in Figure 3A, this comparison revealed an extended interval of significant above-chance decoding during the delay period, i.e., when no stimulus was visually present. This time window in the delay period (860–1,275 ms) was then selected as our temporal region of interest for subsequent delay period decoding analyses. Importantly, however, results using this time period remained the same if decoding accuracies were instead averaged or cluster-corrected across the entire delay period duration (Table S1). Classification was also conducted on the stimulus and response periods, both of which showed periods of accurate decoding across time (Figure S2Aii). Although baseline-correcting each epoch (stimulus/response/delay) individually (with subtracting each epoch's preceding 200 ms) was used to prevent any spill-over across epochs, classification was also performed with baseline-correcting data only once using the pre-stimulus period (i.e., the 200 ms preceding stimulus onset). This approach largely replicated the decoding findings

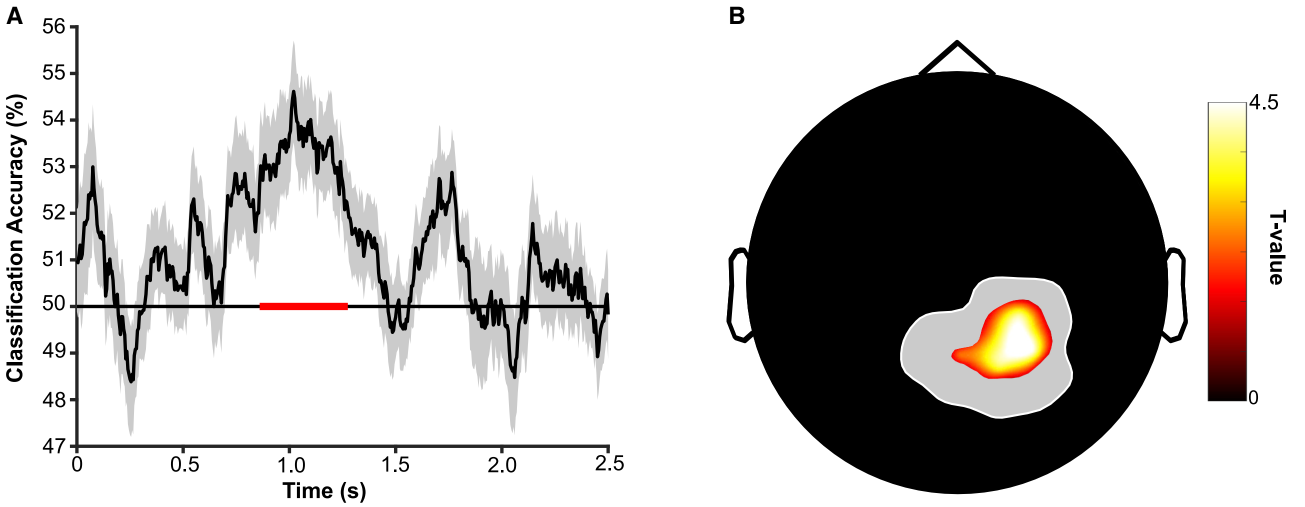

Figure 3. Multivariate decoding of WM representations during the 1-back task

(A) Decoding (mean ± SEM across participants) of object versus scene stimuli across time showed significant above-chance (50%) accuracy in the delay period of the 1-back task (860–1,275 ms into the delay period). The depicted delay period reflects the minimum duration, omitting the variable jitter. The solid red line indicates cluster-corrected significance ($p < 0.05$).

(B) Spatial searchlight decoding during the significant delay period, revealing maximum performance at central-posterior channels (corrected t test versus chance). The gray outline indicates the maximum extent of the searchlight cluster (i.e., including searchlight center and its neighbors; see STAR Methods section for neighbor definition). The full temporal generalization matrix of time-by-time classification and the unthresholded topography is shown in Figure S2.

as before, showing that decoding accuracy was especially pronounced during the stimulus and delay periods of the 1-back task (Figures S2Bi–S2Biii). To further ensure that decodability reflected the goal-directed WM representation of a previously experienced stimulus, we examined the performance of a classifier trained on the stimulus period of the task and tested on the delay period. Above-chance decoding in this case indicates temporal generalization and therefore reactivation of the stimulus-related pattern. Classifier accuracy was indeed significantly greater than chance ($t_{(27)} = 4.85$, $p < 0.001$, Cohen's $d = 0.92$) (Figure S2Aiii).

Previous findings suggest that visual WM content is maintained by posterior sensory rather than frontal executive regions.[44] We thus repeated the classification analysis with a searchlight approach, specifically during the delay period where no visual information was on-screen. Classification performance was assessed for each channel, including its immediate neighbors (mean number of neighbors = 5.7) and focusing on the period that showed maximal decoding across time when including all channels (860–1,275 ms into the delay period; Figure 3A). This approach revealed that stimulus decoding during the delay period was driven largely by central-posterior channels (Figure 3B). Visual inspection of uncorrected significance across the scalp corroborated low accuracy of searchlight decoding using frontal channels (Figure S2C).

Together, these results suggest that stimulus content maintained in WM can be decoded successfully during the delay period of a 1-back task. The central-posterior topography of maximal decodability (despite WM-load-related theta changes over fronto-medial channels; Figure 2B) is consistent with the findings from fMRI[45] and with the notion of frontal theta as an executive control system that does not directly maintain WM content.[20]

Posterior channels are functionally coupled with frontal theta

We next probed whether regions involved in the maintenance of WM content (Figure 3) are coupled to frontal theta rhythms (Figure 2). If theta activity does indeed serve as the mediator between frontal executive and posterior representational regions, one would expect increased coherence between these two regions as a result of increased WM engagement. Consequently, we calculated coherence values between channel Fz (representing the center of the frontal theta cluster previously identified, Figure 2B) and every other channel during the portion of the delay period when content could be significantly decoded (i.e., 860–1,275 ms; Figure 4). Comparison of coherence maps for 1-back versus DMS tasks revealed a significant central-posterior cluster of increased coherence. Of note, and as illustrated in the inset of Figure 4, the resulting cluster overlapped markedly with the results from our searchlight-decoding approach (Figure 3B), suggesting that at least some of the same regions that maintain WM content are coupled to the frontal theta rhythm. Examining the frequency profile of this cluster, it showed maximal coherence at ~7 Hz (results not shown), which matches the peak frequency of the frontal power effect (Figure 2B). A similar cluster (both in frequency and topography) was also present when coherence values were averaged across the full delay period.

To corroborate the regional overlap between (1) coherence with FMT and (2) stimulus category decoding, we first repeated the classification analysis but used only those channels that showed significant coherence in the theta band with frontal regions. Indeed, this approach yielded significant above-chance decoding ($t_{(27)} = 2.70$, $p = 0.01$, Cohen's $d = 0.51$). Second, we divided the cluster of significant coherence with FMT into (1) channels that also show significant stimulus category decoding (including any channel that was a member of a significant

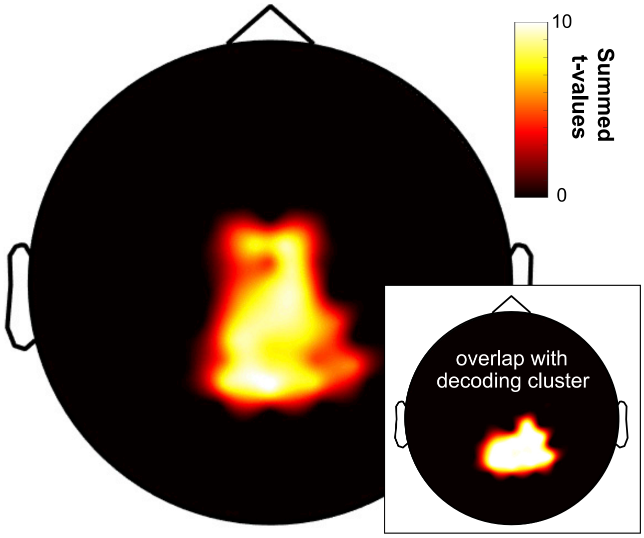

Figure 4. Coupling between posterior "content" channels and frontal theta activity

Taking Fz as the seed channel and comparing 1-back versus DMS task during the time in the delay period when WM content could be significantly decoded (860–1,275 ms) revealed a significant increase in coherence with central-posterior channels in the theta band (4–8 Hz). Main—significant channels, summed across significant frequencies. Inset—overlap with channels contributing to WM content decoding (cf. Figure 3B).

searchlight cluster, as in Figure 3B and the inset of Figure 4) and (2) channels that do not show significant stimulus category decoding. Direct comparison of peak coherence strength with frontal regions (7 Hz) revealed significantly greater coherence for those channels in which we observed significant stimulus category decoding ($t_{(27)} = 2.51$, $p = 0.018$, Cohen's $d = 0.47$). Together, these analyses suggest that functional coupling with frontal regions is particularly enhanced in those posterior regions that represent the maintained stimulus category.

Increasing WM load slows down theta

Given the central role of FMT in coordinating WM, how does an increase in WM load impact theta oscillations? One effect of increased WM load might be an increase in FMT power, perhaps reflecting a greater number and/or level of synchronization of participating neurons.[38,46] Indeed, such power increases have been reported before (e.g., Meltzer et al.[25] and Jensen and Tesche[47]). Another result of increased WM load might be a slowing of the theta rhythm. For instance, the Jensen and Lisman model[15,17] holds that the ongoing theta cycle governs the serial reactivation item-coding cell assemblies. Thus, the duration of a given theta cycle is the limiting factor in how many items can be successfully maintained. A slowing in frequency would therefore facilitate the maintenance additional items within the same theta cycle. In our study, we tested the effect of increased WM load on FMT by comparing the 1-back task with a 2-back variant (Figure 1).

Behaviorally, participants continued to show high accuracy in the 2-back task, although, as with the 1-back task, accuracy was significantly lower in the 2-back relative to the DMS task ($t_{(27)} = 3.36$, $p = 0.002$, Cohen's $d = 0.63$). Although there was no significant difference in the accuracy between the 2-back and the 1-back tasks ($t_{(27)} = 0.816$, $p = 0.42$, Cohen's $d = 0.15$), the increase in WM load did induce a significant slowing of reaction times (RTs) (paired-samples t test of 2-back versus 1-back; $t_{(27)} = 3.85$, $p < 0.001$, Cohen's $d = 0.73$).

We first examined the effects of load (1-back versus 2-back) and frequency (between 4 and 8 Hz in 0.5 Hz increments) on power within the theta band over the frontal-medial cluster. Despite previous reports of theta power scaling with WM load,[25,47] we did not find strong evidence for a theta power increase from the 1-back to the 2-back task in the present data. Examining FMT power (averaged across the delay period) via a repeated-measures ANOVA revealed no significant main effect of load ($F_{(1,27)} = 3.26$, $p = 0.082$, $\eta^2 = 0.12$). Unsurprisingly, given the 1/f component present in EEG data,[48] there was a main effect of frequency ($F_{(8,27)} = 7.56$, $p < 0.001$, $\eta^2 = 0.28$). Importantly, however, there was also a significant interaction between frequency and load ($F_{(8,216)} = 9.81$, $p < 0.001$, $\eta^2 = 0.36$). Follow-up paired t tests demonstrated that this was driven by a relative power increase in the 2-back condition in the lower theta range (at the lower end of the theta range at frequencies between 4 and 5.5 Hz (4 Hz, $t_{(27)} = 3.29$, $p_{Holm} = 0.024$, Cohen's $d = 0.62$; 4.5 Hz, $t_{(27)} = 3.53$, $p_{Holm} = 0.018$, Cohen's $d = 0.67$; 5 Hz, $t_{(27)} = 3.07$, $p_{Holm} = 0.035$, Cohen's $d = 0.58$), with this difference diminished at higher frequencies (5.5 Hz, $t_{(27)} = 2.72$, $p_{Holm} = 0.066$, Cohen's $d = 0.51$; 6 Hz, $t_{(27)} = 2.41$, $p_{Holm} = 0.12$, Cohen's $d = 0.46$; 6.5 Hz, $t_{(27)} = 1.82$, $p = 0.32$, Cohen's $d = 0.34$; 7 Hz, $t_{(27)} = 0.63$, $p_{Holm} = 1.06$, Cohen's $d = 0.12$; 7.5 Hz, $t_{(27)} = -0.54$, $p_{Holm} = 0.60$, Cohen's $d = 0.10$; 8 Hz, $t_{(27)} = -1.17$, $p_{Holm} = 0.76$, Cohen's $d = 0.22$)).

To confirm that this change in frequency was indeed a slowing (and thus broadening) of theta oscillations, we defined, for each participant and n-back condition, the peak theta frequency (4–8 Hz) during the delay period. For every trial and for every participant, the frequency at which the most prominent peak in the spectrum occurred was taken. These peak values were averaged by the condition resulting in an average theta peak for each condition for each participant. Consistent with the Jensen and Lisman model, a paired t test revealed a small but highly consistent decrease in peak frequency between the 1-back and 2-back tasks (means: 5.85 Hz versus 5.77 Hz; $t_{(27)} = 5.02$, $p < 0.001$, Cohen's $d = 0.95$; Figure 5). This peak detection approach should be largely insensitive to the 1/f component of EEG signals, but to ensure that this was the case, and given the possible functional significance of a change in this exponent,[48] the same method was applied to data to which the irregular resampling auto-spectral analysis (IRASA) algorithm had been applied.[49] The significant slowing effect persisted (means: 6.05 Hz versus 5.99 Hz; $t_{(27)} = 2.84$, $p = 0.008$, Cohen's $d = 0.54$). Finally, to rule out the possibility that this finding had occurred due to any volatility in calculating power on the single-trial level, the individual trial spectra were smoothed by sliding average (variable window; 0.20–1.00 Hz). Again, significant slowing for 2-back versus 1-back was observed for all smoothing ranges (t values ≥ 4.24, p values < 0.001, Cohen's d values ≥ 0.80).

If FMT (and its synchrony with posterior regions that maintain content) is indeed a principal mechanism of WM function, frontal-posterior coherence in the theta band should not only be evident in the 2-back task but also show a commensurate slowing

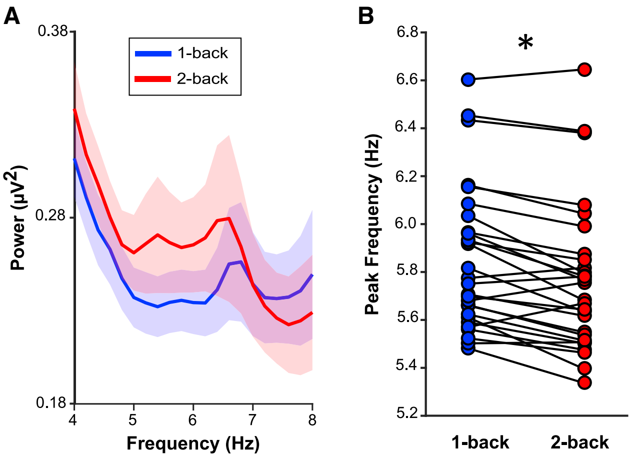

Figure 5. Theta frequency slows to accommodate an additional WM item

(A) Raw mean (± SEM) power values in the theta band (4–8 Hz) across participants, averaged across the delay periods of the 1-back task (blue) and the 2-back task (red). Note the relative shift toward lower frequencies for the 2-back task.

(B) Theta peak frequencies for 1-back and 2-back delay periods shown for each participant. Asterisk indicates statistical significance at $\alpha = 0.05$.

in frequency. Comparison of the 2-back task with the DMS task indeed revealed a significant central-posterior cluster of increased coherence (Figure S4A [inset]), similar to that observed when comparing the 1-back task with the DMS task. Importantly, subtracting coherence values in the 1-back task from the 2-back task revealed a relative increase during 2-back maintenance at the lower end of the theta band (Figure S4B), peaking at 5 Hz ($t_{(27)} = 2.36$, $p = 0.026$, Cohen's $d = 0.45$).

DISCUSSION

Our study elucidates the dynamic interplay between the two key components of WM, i.e., an executive control mechanism and the representation of stimulus content. Using a paradigm that manipulated WM demand (Figure 1), we found a power increase in FMT during 1-back tasks relative to a DMS task (Figure 2). Multivariate pattern analysis showed that WM content, i.e., whether an individual maintained an object or a scene image, could be decoded successfully during the delay period of the 1-back task from central-posterior channels (rather than from those channels showing the theta power effects; Figure 3). Importantly, channels that contributed to decoding also showed increased coherence (in the delay period of the 1-back task versus the DMS task) in the theta frequency band with frontal sites sensitive to WM load. Lastly, comparing the 2-back task with the 1-back task, we found that maintaining an additional item in WM leads to slowing of the FMT rhythm (Figure 5), consistent with computational accounts suggesting a broadening of the theta cycle to accommodate multiple WM items.

The finding that WM-induced FMT also governed functional coupling with posterior channels that are most informative to decoding strongly points to a role of FMT in coordinating posterior WM maintenance. This observation unifies a series of recent findings and highlights the importance of theta oscillations as the interlocutor between regional WM sub-systems. Although WM content has been shown to be preferentially decoded from posterior regions, there has been a paucity of evidence to directly associate a measure of WM content with theta activity from frontal regions. That is, there is considerable evidence of FMT as an executive control system in WM[21,31] and of posterior localization of WM content.[45,50] However, there is little extant evidence directly connecting WM content with frontal theta activity. Previous work has largely focused on how frontal theta interacts either with activity in other frequency bands, e.g., gamma activity[51] or with neuronal spiking,[18,52] neither of which provide a direct readout of high-level WM content. A recent study, which did measure WM content, observed modulation of decoding from posterior regions according to a theta/alpha rhythm[53] but did not link this pattern to frontal activity.

The topographical dissociation of frontal control mechanisms (Figure 2B) versus posterior content maintenance (Figure 3B) dovetails with WM models proposing a domain-general role for prefrontal cortex in executive control.[20] According to the sensory recruitment model, the direct maintenance of content is then accomplished by posterior regions,[54] specifically those that are involved in the processing of the stimulus in a non-WM context. It deserves mention though that other studies have reported content-related WM activity in frontal regions (e.g., Riley and Constantinidis [55], Meyer et al.[56], and Lee and Baker[57]). However, decoding from frontal regions has been proposed to reflect a transformed representation, perhaps representing goal states or action plans rather than stimulus content per se.[58,59] In the present study, the motor response (''match''/''non-match'') was orthogonal to the stimulus category (''object''/''scene'') on which the classifier was trained.

To what extent do our decoding results reflect actual WM content? Although the behavioral task prescribed maintenance of a specific item representation, we use here the superordinate category (i.e., object versus scene) in our classification regimen. The rationale for this approach is that, although participants most likely indeed maintain individual exemplars (as the labels ''object'' and ''scene'' would be insufficient to solve the task), multivariate decoding greatly benefits from higher within-category than between-category similarity of exemplars (i.e., objects being more similar to other objects than to scenes and vice versa).[60] The same approach has been used in long-term memory research, where greater decodability of superordinate stimulus categories is harnessed as a proxy for recall of individual items.[61,62] Likewise, we cannot ascertain which particular features drive decodability of objects versus scenes (lower-level perceptual versus higher-level conceptual). Importantly though, using temporal generalization, we show that the same features that allow discrimination of objects versus scenes during stimulus encoding are reinstated during the maintenance period (Figure S2), tethering decoded WM content to preceding stimulus perception.

In any case, could decodability of WM content during the delay period reflect a spill-over from the preceding stimulus or response interval? In a recent EEG study in which orientations of a teardrop shape were decoded, there was an initial increase in accuracy after stimulus offset followed by a sustained decline.[63] This pattern more closely resembles the response period in our dataset (Figure S2) and is consistent with the finding

that decodability rebounds after stimulus offset.[64] However, stimulus presentation and maintenance were separated by a minimum of 750 ms in our paradigm (Figure 1), and maximum decodability was actually seen from 860 to 1,275 ms after delay onset, mitigating the impacts of preceding stimulus or response windows. Further precluding this explanation is that prior to classification, data were baseline corrected to the immediately preceding 200 ms (i.e., the final 200 ms of the response period was subtracted from the delay period).

Assuming that a frontal executive does coordinate WM maintenance via theta oscillations, how does this system respond to increasing task demand? One possibility is scaling of theta power. Theta power is frequently greater in conditions in which more items must be stored in WM.[21,65] Furthermore, parametric theta power scaling with conditions of increasing load has previously been observed,[25,47] although not without exception. Payne and Kounios[32] systematically varied load by presenting 2, 4, or 6 letters, observing an increase in fronto-parietal coherence but not in theta power. Many of these studies employed Sternberg-like paradigms, but studies more comparable with the current paradigm also show some inconsistencies. Brookes et al.[39] observed robust theta power increases between 0-, 1-, and 2-back tasks, whereas Missonnier et al.[66] did not find a significant difference between 1- and 2-back conditions. The extent to which the effect of load is influenced by other differences between paradigms, such as stimulus complexity, block length, or delay duration, is important to address in future work. In the present study, although FMT power was greater in the 1-back relative to the DMS task, this increase did not extend to the 2-back task.

An alternative way FMT might respond to WM load is a change in frequency. Indeed, in place of scaling of FMT power, we provide here the first empirical evidence of slowing of the FMT rhythm in response to increasing WM load. Importantly, load here refer to the increase in the number of items needing to be remembered. Thus, this may reflect both the direct maintenance of an additional item and the increased need for control of these representations. The magnitude of slowing was moderate but highly consistent across participants. According to the Jensen-Lisman model,[15,17] slowing of the carrier theta frequency facilitates bursting of additional item-coding cell assemblies in each cycle while maintaining phase separation among items. Although a specific slowing in FMT has not previously been demonstrated, Axmacher et al.[67] did observe, in intracranial hippocampal recordings during a WM (Sternberg) task, a load-dependent reduction of the theta frequency modulating power in the beta/low gamma band. Further indirect evidence for load-dependent theta slowing comes from a series of studies employing tACS. Modulating the speed of endogenous theta by means of stimulating at a low (3 Hz) or high (7 Hz) theta frequency was shown to improve or impede WM function, respectively.[68–70] The data here are in agreement with the implication of these stimulation studies—although theta power is critical for WM (as evidenced by the increase in the 1-back relative to the DMS task), the limiting factor in holding multiple items may in fact be the frequency of ongoing theta oscillations. The importance of theta frequency/phase is further supported by recent evidence of phase coding in the human medial temporal lobe. Stimulus-specific cell firing patterns show theta phase precession, whereby stimuli-coding firing occurs at earlier theta phases according to a stimulus position in a sequence.[71] Additionally, when multiple items were maintained in WM, whether a stimulus was in memory or not could be determined by the theta phase to which the relevant cells' firing locked.[72] Nevertheless, the exact functional roles of theta power versus theta phase in WM maintenance should be more systematically explored in future work. In the present case, we observed a change in theta power in response to increased executive WM demand (1-back versus DMS task) and a change in frequency in response to an additional to-be-remembered item (2-back versus 1-back). The use of additional load levels in the n-back task, for example, would further clarify the limits of frequency slowing and whether frequency slowing occurs in lieu of, or in addition to, an increase in power.

To summarize, we show that frontal theta rhythms orchestrate the maintenance of stimulus representations in posterior brain regions in the service of WM performance. Increasing the amount of information to be maintained led to a slowing of theta frequency, consistent with the idea that longer duty cycles are needed to accommodate additional items held in WM.

STAR★METHODS

Detailed methods are provided in the online version of this paper and include the following:

- KEY RESOURCES TABLE
- RESOURCE AVAILABILITY
  - Lead contact
  - Materials availability
  - Data and code availability
- EXPERIMENTAL MODEL AND SUBJECT DETAILS
- METHOD DETAILS
  - Procedure
  - Experimental design and statistical analysis
  - EEG setup and pre-processing
  - Time-frequency calculations
  - Classification
  - Connectivity
- QUANTIFICATION AND STATISTICAL ANALYSIS
  - Subject exclusion criterion
  - Inferential statistics

SUPPLEMENTAL INFORMATION

Supplemental information can be found online at https://doi.org/10.1016/j.cub.2022.03.045.

ACKNOWLEDGMENTS

This work was supported by a Wellcome Trust/Royal Society Sir Henry Dale Fellowship (107672/Z/15/Z) to B.P.S., an ERC Consolidator grant (101001121) to B.P.S., and an MRC IMPACT PhD studentship awarded to O.R. (1973157).

AUTHOR CONTRIBUTIONS

O.R. designed research, performed research, analyzed data, and wrote the paper. K.S. designed research and wrote the paper. B.P.S. designed research and wrote the paper.

DECLARATION OF INTERESTS

The authors declare no competing interests.

Received: June 2, 2021
Revised: October 11, 2021
Accepted: March 15, 2022
Published: April 5, 2022

REFERENCES

1. The Mathworks Inc. MATLAB and Statistics Toolbox. https://www.mathworks.com/products/matlab.html.
2. Brainard, D.H. (1997). The psychophysics toolbox. Spat. Vis. *10*, 433–436.
3. Kleiner, M., Brainard, D.H., Pelli, D.G., Broussard, C., Wolf, T., and Niehorster, D. (2007). What's new in psychtoolbox-3? Perception *36*, 1–16.
4. R Core Team (2018). A language and environment for statistical computing (R Foundation for Staistical Computing).
5. Oostenveld, R., Fries, P., Maris, E., and Schoffelen, J.M. (2011). FieldTrip: open source software for advanced analysis of MEG, EEG, and invasive electrophysiological data. Comput. Intell. Neurosci. *2011*, 156869.
6. Kearney, K. (2019). boundedline.m: line with shaded error/confidence bounds (GitHub). https://github.com/kakearney/boundedline-pkg.
7. Baddeley, A. (1992). Working memory. Science *255*, 556–559.
8. Baddeley, A. (2003). Working memory: looking back and looking forward. Nat. Rev. Neurosci. *4*, 829–839.
9. Imaruoka, T., Saiki, J., and Miyauchi, S. (2005). Maintaining coherence of dynamic objects requires coordination of neural systems extended from anterior frontal to posterior parietal brain cortices. Neuroimage *26*, 277–284.
10. Bressler, S.L., and Menon, V. (2010). Large-scale brain networks in cognition: emerging methods and principles. Trends Cogn. Sci. *14*, 277–290.
11. Vogel, E.K., and Machizawa, M.G. (2004). Neural activity predicts individual differences in visual working memory capacity. Nature *428*, 748–751.
12. Owen, A.M., McMillan, K.M., Laird, A.R., and Bullmore, E. (2005). N-back working memory paradigm: a meta-analysis of normative functional neuroimaging studies. Hum. Brain Mapp. *25*, 46–59.
13. Sauseng, P., Griesmayr, B., Freunberger, R., and Klimesch, W. (2010). Control mechanisms in working memory: a possible function of EEG theta oscillations. Neurosci. Biobehav. Rev. *34*, 1015–1022.
14. Von Stein, A., and Sarnthein, J. (2000). Different frequencies for different scales of cortical integration: from local gamma to long range alpha/theta synchronization. Int. J. Psychophysiol. *38*, 301–313.
15. Lisman, J.E., and Jensen, O. (2013). The theta-gamma neural code. Neuron *77*, 1002–1016.
16. Lisman, J.E., and Idiart, M.A.P. (1995). Storage of 7 ± 2 short-term memories in oscillatory subcycles. Science *267*, 1512–1515.
17. Jensen, O., and Lisman, J.E. (1998). An oscillatory short-term memory buffer model can account for data on the Sternberg task. J. Neurosci. *18*, 10688–10699.
18. Siegel, M., Warden, M.R., and Miller, E.K. (2009). Phase-dependent neuronal coding of objects in short-term memory. Proc. Natl. Acad. Sci. USA *106*, 21341–21346.
19. Bahramisharif, A., Jensen, O., Jacobs, J., and Lisman, J. (2018). Serial representation of items during working memory maintenance at letter-selective cortical sites. PLoS Biol. *16*, e2003805.
20. D'Esposito, M., and Postle, B.R. (2015). The cognitive neuroscience of working memory. Annu. Rev. Psychol. *66*, 115–142.
21. Hsieh, L.T., and Ranganath, C. (2014). Frontal midline theta oscillations during working memory maintenance and episodic encoding and retrieval. Neuroimage *85*, 721–729.
22. Michels, L., Bucher, K., Lüchinger, R., Klaver, P., Martin, E., Jeanmonod, D., and Brandeis, D. (2010). Simultaneous EEG-fMRI during a working memory task: modulations in low and high frequency bands. PLoS One *5*, e10298.
23. Raghavachari, S., Kahana, M.J., Rizzuto, D.S., Caplan, J.B., Kirschen, M.P., Bourgeois, B., Madsen, J.R., and Lisman, J.E. (2001). Gating of human theta oscillations by a working memory task. J. Neurosci. *21*, 3175–3183.
24. Cavanagh, J.F., and Frank, M.J. (2014). Frontal theta as a mechanism for cognitive control. Trends Cogn. Sci. *18*, 414–421.
25. Meltzer, J.A., Zaveri, H.P., Goncharova, I.I., Distasio, M.M., Papademetris, X., Spencer, S.S., Spencer, D.D., and Constable, R.T. (2008). Effects of working memory load on oscillatory power in human intracranial EEG. Cereb. Cortex *18*, 1843–1855.
26. Tsujimoto, T., Shimazu, H., and Isomura, Y. (2006). Direct recording of theta oscillations in primate prefrontal and anterior cingulate cortices. J. Neurophysiol. *95*, 2987–3000.
27. Mitchell, D.J., McNaughton, N., Flanagan, D., and Kirk, I.J. (2008). Frontal-midline theta from the perspective of hippocampal "theta". Prog. Neurobiol. *86*, 156–185.
28. Maurer, U., Brem, S., Liechti, M., Maurizio, S., Michels, L., and Brandeis, D. (2015). Frontal midline theta reflects individual task performance in a working memory task. Brain Topogr. *28*, 127–134.
29. Brzezicka, A., Kamiński, J., Reed, C.M., Chung, J.M., Mamelak, A.N., and Rutishauser, U. (2019). Working memory load-related theta power decreases in dorsolateral prefrontal cortex predict individual differences in performance. J. Cogn. Neurosci. *31*, 1290–1307.
30. Zakrzewska, M.Z., and Brzezicka, A. (2014). Working memory capacity as a moderator of load-related frontal midline theta variability in Sternberg task. Front. Hum. Neurosci. *8*, 399.
31. Riddle, J., Scimeca, J.M., Cellier, D., Dhanani, S., and D'Esposito, M. (2020). Causal evidence for a role of theta and alpha oscillations in the control of working memory. Curr. Biol. *30*, 1748.e4–1754.e4.
32. Payne, L., and Kounios, J. (2009). Coherent oscillatory networks supporting short-term memory retention. Brain Res. *1247*, 126–132.
33. Johnson, E.L., Dewar, C.D., Solbakk, A.K., Endestad, T., Meling, T.R., and Knight, R.T. (2017). Bidirectional frontoparietal oscillatory systems support working memory. Curr. Biol. *27*, 1829.e4–1835.e4.
34. Parto Dezfouli, M., Davoudi, S., Knight, R.T., Daliri, M.R., and Johnson, E.L. (2021). Prefrontal lesions disrupt oscillatory signatures of spatiotemporal integration in working memory. Cortex *138*, 113–126.
35. Reinhart, R.M.G., and Nguyen, J.A. (2019). Working memory revived in older adults by synchronizing rhythmic brain circuits. Nat. Neurosci. *22*, 820–827.
36. Polanía, R., Nitsche, M.A., Korman, C., Batsikadze, G., and Paulus, W. (2012). The importance of timing in segregated theta phase-coupling for cognitive performance. Curr. Biol. *22*, 1314–1318.
37. Sauseng, P., Klimesch, W., Doppelmayr, M., Hanslmayr, S., Schabus, M., and Gruber, W.R. (2004). Theta coupling in the human electroencephalogram during a working memory task. Neurosci. Lett. *354*, 123–126.
38. Buzsáki, G., and Draguhn, A. (2004). Neuronal oscillations in cortical networks. Science *304*, 1926–1929.
39. Brookes, M.J., Wood, J.R., Stevenson, C.M., Zumer, J.M., White, T.P., Liddle, P.F., and Morris, P.G. (2011). Changes in brain network activity during working memory tasks: a magnetoencephalography study. Neuroimage *55*, 1804–1815.
40. Hsieh, L.T., Ekstrom, A.D., and Ranganath, C. (2011). Neural oscillations associated with item and temporal order maintenance in working memory. J. Neurosci. *31*, 10803–10810.
41. Zuure, M.B., Hinkley, L.B.N., Tiesinga, P.H.E., Nagarajan, S.S., and Cohen, M.X. (2020). Multiple midfrontal thetas revealed by source separation of simultaneous MEG and EEG. J. Neurosci. *40*, 7702–7713.
42. Onton, J., Delorme, A., and Makeig, S. (2005). Frontal midline EEG dynamics during working memory. Neuroimage *27*, 341–356.

43. Cohen, J. (1992). A power primer. Psychol. Bull. *112*, 155–159.
44. Sreenivasan, K.K., Curtis, C.E., and D'Esposito, M. (2014). Revisiting the role of persistent neural activity during working memory. Trends Cogn. Sci. *18*, 82–89.
45. Harrison, S.A., and Tong, F. (2009). Decoding reveals the contents of visual working memory in early visual areas. Nature *458*, 632–635.
46. Buzsáki, G., Anastassiou, C.A., and Koch, C. (2012). The origin of extracellular fields and currents—EEG, ECoG, LFP and spikes. Nat. Rev. Neurosci. *13*, 407–420.
47. Jensen, O., and Tesche, C.D. (2002). Frontal theta activity in humans increases with memory load in a working memory task. Eur. J. Neurosci. *15*, 1395–1399.
48. Donoghue, T., Haller, M., Peterson, E.J., Varma, P., Sebastian, P., Gao, R., Noto, T., Lara, A.H., Wallis, J.D., Knight, R.T., et al. (2020). Parameterizing neural power spectra into periodic and aperiodic components. Nat. Neurosci. *23*, 1655–1665.
49. Wen, H., and Liu, Z. (2016). Separating fractal and oscillatory components in the power spectrum of neurophysiological signal. Brain Topogr. *29*, 13–26.
50. Christophel, T.B., Hebart, M.N., and Haynes, J.D. (2012). Decoding the contents of visual short-term memory from human visual and parietal cortex. J. Neurosci. *32*, 12983–12989.
51. Berger, B., Griesmayr, B., Minarik, T., Biel, A.L., Pinal, D., Sterr, A., and Sauseng, P. (2019). Dynamic regulation of interregional cortical communication by slow brain oscillations during working memory. Nat. Commun. *10*, 4242.
52. Crowe, D.A., Goodwin, S.J., Blackman, R.K., Sakellaridi, S., Sponheim, S.R., MacDonald, A.W., and Chafee, M.V. (2013). Prefrontal neurons transmit signals to parietal neurons that reflect executive control of cognition. Nat. Neurosci. *16*, 1484–1491.
53. Ten Oever, S., De Weerd, P., and Sack, A.T. (2020). Phase-dependent amplification of working memory content and performance. Nat. Commun. *11*, 1832.
54. Scimeca, J.M., Kiyonaga, A., and D'Esposito, M. (2018). Reaffirming the sensory recruitment account of working memory. Trends Cogn. Sci. *22*, 190–192.
55. Riley, M.R., and Constantinidis, C. (2015). Role of prefrontal persistent activity in working memory. Front. Syst. Neurosci. *9*, 181.
56. Meyer, T., Qi, X.L., Stanford, T.R., and Constantinidis, C. (2011). Stimulus selectivity in dorsal and ventral prefrontal cortex after training in working memory tasks. J. Neurosci. *31*, 6266–6276.
57. Lee, S.H., and Baker, C.I. (2016). Multi-voxel decoding and the topography of maintained information during visual working memory. Front. Syst. Neurosci. *10*, 2.
58. Christophel, T.B., Klink, P.C., Spitzer, B., Roelfsema, P.R., and Haynes, J.D. (2017). The distributed nature of working memory. Trends Cogn. Sci. *21*, 111–124.
59. Lee, S.H., Kravitz, D.J., and Baker, C.I. (2013). Goal-dependent dissociation of visual and prefrontal cortices during working memory. Nat. Neurosci. *16*, 997–999.
60. Norman, K.A., Polyn, S.M., Detre, G.J., and Haxby, J.V. (2006). Beyond mind-reading: multi-voxel pattern analysis of fMRI data. Trends Cogn. Sci. *10*, 424–430.
61. Polyn, S.M., Natu, V.S., Cohen, J.D., and Norman, K.A. (2005). Neuroscience: category-specific cortical activity precedes retrieval during memory search. Science *310*, 1963–1966.
62. Jafarpour, A., Fuentemilla, L., Horner, A.J., Penny, W., and Duzel, E. (2014). Replay of very early encoding representations during recollection. J. Neurosci. *34*, 242–248.
63. Bae, G.Y., and Luck, S.J. (2018). Dissociable decoding of spatial attention and working memory from EEG oscillations and sustained potentials. J. Neurosci. *38*, 409–422.
64. Robinson, A.K., Grootswagers, T., and Carlson, T.A. (2019). The influence of image masking on object representations during rapid serial visual presentation. Neuroimage *197*, 224–231.
65. Gevins, A., Smith, M.E., McEvoy, L., and Yu, D. (1997). High-resolution EEG mapping of cortical activation related to working memory: effects of task difficulty, type of processing, and practice. Cereb. Cortex *7*, 374–385.
66. Missonnier, P., Deiber, M.P., Gold, G., Millet, P., Gex-Fabry Pun, M., Fazio-Costa, L., Giannakopoulos, P., and Ibáñez, V. (2006). Frontal theta event-related synchronization: comparison of directed attention and working memory load effects. J. Neural Transm. (Vienna) *113*, 1477–1486.
67. Axmacher, N., Henseler, M.M., Jensen, O., Weinreich, I., Elger, C.E., and Fell, J. (2010). Cross-frequency coupling supports multi-item working memory in the human hippocampus. Proc. Natl. Acad. Sci. USA *107*, 3228–3233.
68. Bender, M., Romei, V., and Sauseng, P. (2019). Slow theta tACS of the right parietal cortex enhances contralateral visual working memory capacity. Brain Topogr. *32*, 477–481.
69. Wolinski, N., Cooper, N.R., Sauseng, P., and Romei, V. (2018). The speed of parietal theta frequency drives visuospatial working memory capacity. PLoS Biol. *16*, e2005348.
70. Vosskuhl, J., Huster, R.J., and Herrmann, C.S. (2015). Increase in short-term memory capacity induced by down-regulating individual theta frequency via transcranial alternating current stimulation. Front. Hum. Neurosci. *9*, 257.
71. Reddy, L., Self, M.W., Zoefel, B., Poncet, M., Possel, J.K., Peters, J.C., Baayen, J.C., Idema, S., VanRullen, R., and Roelfsema, P.R. (2021). Theta-phase dependent neuronal coding during sequence learning in human single neurons. Nat. Commun. *12*, 4839.
72. Kamiński, J., Brzezicka, A., Mamelak, A.N., and Rutishauser, U. (2020). Combined phase-rate coding by persistently active neurons as a mechanism for maintaining multiple items in working memory in humans. Neuron *106*, 256.e3–264.e3.
73. Scheeringa, R., Petersson, K.M., Oostenveld, R., Norris, D.G., Hagoort, P., and Bastiaansen, M.C.M. (2009). Trial-by-trial coupling between EEG and BOLD identifies networks related to alpha and theta EEG power increases during working memory maintenance. Neuroimage *44*, 1224–1238.
74. Brodeur, M.B., Dionne-Dostie, E., Montreuil, T., and Lepage, M. (2010). The bank of standardized stimuli (BOSS), a new set of 480 normative photos of objects to be used as visual stimuli in cognitive research. PLoS One *5*, e10773.
75. Xiao, J., Hays, J., Ehinger, K.A., Oliva, A., and Torralba, A. (2010). SUN database: large-scale scene recognition from abbey to zoo. In Proceedings of the IEEE Computer Society Conference on Computer Vision and Pattern Recognition (IEEE), pp. 3485–3492.
76. Treder, M.S. (2020). MVPA-light: a classification and regression toolbox for multi-dimensional data. Front. Neurosci. *14*, 289.
77. Grootswagers, T., Wardle, S.G., and Carlson, T.A. (2017). Decoding dynamic brain patterns from evoked responses: a tutorial on multivariate pattern analysis applied to time series neuroimaging data. J. Cogn. Neurosci. *29*, 677–697.
78. Bastos, A.M., and Schoffelen, J.M. (2015). A tutorial review of functional connectivity analysis methods and their interpretational pitfalls. Front. Syst. Neurosci. *9*, 175.
79. Maris, E., and Oostenveld, R. (2007). Nonparametric statistical testing of EEG- and MEG-data. J. Neurosci. Methods *164*, 177–190.
80. Holm, S. (1979). A simple sequentially rejective multiple test procedure. Scand. J. Stat. *6*, 65–70.

STAR★METHODS

KEY RESOURCES TABLE

REAGENT or RESOURCE	SOURCE	IDENTIFIER
Deposited data		
OSF	https://osf.io/ub9k6/	ub9k6
Software and algorithms		
MATLAB	https://uk.mathworks.com/products/matlab.html	2016b
Fieldtrip	https://www.fieldtriptoolbox.org/	20210308
MVPA-light	https://github.com/treder/MVPA-Light	N/A
Psychophysics Toolbox	http://psychtoolbox.org	Version 3 (PTB-3)
Rstudio	https://www.rstudio.com/	4.0.2

RESOURCE AVAILABILITY

Lead contact

For further information or requests, contact should be directed towards the lead contact, Bernhard Staresina (bernhard.staresina@psy.ox.ac.uk)

Materials availability

This study did not generate new, unique reagents or materials.

Data and code availability

The analysis scripts & processed data are available at https://osf.io/ub9k6/.

All behavioural tasks were created and presented using Matlab 2016b[1] and Psychtoolbox (Version 3.0.16).[2,3] Analyses were conducted with custom Matlab and R scripts (Version 3.4.3).[4] EEG analyses were performed with Fieldtrip functions (Version 20210308).[5] Plots also made use of *boundedline*.[6]

EXPERIMENTAL MODEL AND SUBJECT DETAILS

Thirty-three participants in total were tested. All participants gave written informed consent and all procedures were approved by the University of Birmingham Ethics Committee. Participants were right-handed, aged between 18 and 35, and had no history of psychological or neurological disorder. Data from two participants were removed for low behavioural performance (see "quantification and statistical analysis" section for further detail). Data from a further three participants were removed due to poor EEG quality. All analyses therefore focussed on the remaining 28 participants (18 female, mean age of 22.64 years, SD = 3.95, range = 18-33). This sample size provides 80% power to detect a Cohen's d effect size of >= 0.55. WM-induced FMT effects have been observed with similar or smaller sample sizes (e.g.,[47,73]).

METHOD DETAILS

Procedure

Two behavioural tasks were employed in this experiment: a delayed-match-to-sample (DMS) task and an n-back task featuring two levels of working memory load. Stimuli were 350x350 pixel colour images of one of three categories: object, face, or scene. There were five unique stimuli from each category. The DMS task included all three categories, whereas the n-back task used only the object and the scene stimuli. The additional category in the DMS task was included to facilitate alternative analyses outside the scope of the results we report here. The stimuli were obtained from the BOSS[74] and SUN[75] online databases.

After EEG setup was complete, participants performed the first run of the DMS task. Within each run, each unique image (to-be-compared to the probe) was presented six times. Across both runs of the DMS task, each stimulus was therefore presented 12 times. Given the 15 unique stimuli presented, this resulted in a total of 180 trials across the DMS task. For each trial a randomly selected probe image was presented. Following completion of the DMS task, participants completed the n-back task, which consisted of 12 blocks (8x2-back; 4x1-back). Each block contained 36+n trials. At the beginning and the end of each of these blocks, a fixation cross was present on the screen and participants were instructed to focus on the cross and to think of nothing in particular. This period served as a cognitive baseline. A pre-stimulus fixation period was not employed during the task because in the n-back task trials were not discrete. Specifically, any one item needed to be maintained from one trial to another and so there was no time at which participants

were not required to hold stimulus content in WM. After completion of the n-back task, participants performed the second run of the DMS task in which each stimulus was again presented six times in a random order. The two runs were performed before and after the n-back task to account for any changes in the EEG signal across the recording session (e.g., signal drift).

Experimental design and statistical analysis

The experimental tasks are illustrated in Figure 1. In the Delayed-match-to-sample (DMS) task, participants were asked to focus on a central fixation cross before an image of an object, scene, or face was presented for $\geq$750 ms. After a delay period of $\geq$2500 ms, a probe stimulus (randomly selected from the full stimulus set) was shown for $\geq$750 ms. In the subsequent response window, an 'X' was present on the screen for 750 ms and participants responded using either the left or right arrow key (counter-balanced across participants) to indicate whether the probe's identity was the same as the first image presented in the trial (i.e., whether it was a 'match' or 'non-match'). In both cases, participants were required to make a response. Here and in the n-back task, identity refers to the unique stimulus. Thus although stimuli of different categories were employed in both tasks, the category of any stimulus had no bearing on the task the participant had been instructed to perform. The duration of the initial stimulus, delay period, and the probe stimulus were all jittered so that trials lasted for the base duration plus 0, 50, 100, or 150 ms. Each trial's temporal jitter was randomly assigned ensuring that each jitter possibility (including no jitter) was equally represented in each block independent of category.

In the n-back task, participants were presented with an image of an object or a scene for $\geq$750 ms. An 'X' then appeared on the screen for 750 ms during which participants were required to respond 'match' or 'non-match' with either the left or right arrow key (counter-balanced across participants) to indicate whether the identity of stimulus just seen matched that of the stimulus seen *n* trials back. As before, identity here refers to a singular stimulus meaning that the category of a given stimulus (object/scene) was orthogonal to the task the participants were required to perform. A '+' was then presented for $\geq$2500 ms. Participants were required to maintain the relevant stimulus (1-back) or stimuli (2-back), so that they could make the n-back match/non-match judgement on the following trial. The stimulus and delay periods were jittered by 0, 50, 100, or 150 ms. As with the DMS task, the possible jitter options were balanced within blocks independent of stimulus category.

EEG setup and pre-processing

EEG data were collected using a BioSemi system with 128 channels at a 1024 Hz sampling rate. Data were re-referenced offline to the average of the two mastoids. Eye blinks were removed from data using independent components analysis, as implemented by 'runica' in Fieldtrip's *ft_componentanalysis*. Consistently noisy channels were interpolated using a weighted average of neighbours. Data were then high-pass filtered at 0.3 Hz prior to all other analyses.

Time-frequency calculations

Time-frequency spectra were calculated using Fieldtrip's *mtmconvol* function. Power in frequencies from 2 to 10 Hz (0.5 Hz steps) were computed across the delay period using a Hanning taper. Power was resolved in 50 ms increments. Data were convolved with a variable number of cycles per frequency band. Two cycles were used for frequencies 2-3.5 Hz; 3 cycles for 4-4.5 Hz; 4 cycles for 5-5.5 Hz; and 5 cycles for 6-10 Hz. These power values were averaged across the full delay period and compared between the 1-back task and the DMS task.

Following this analysis, we assessed whether there was a difference in the FMT peak frequency for 1-back vs. 2-back tasks. To this end, spectral power was calculated for all channels that were members of the frontal cluster previously identified. Power was computed for frequencies between 4-8 Hz in 0.2 Hz increments across the full delay period for both conditions for each of these channels. For this analysis, power was calculated via Fieldtrip's *mtmfft* function. Power spectra from individual channels were then averaged. Thus, for the resulting power spectrum of each participant and every trial, local maxima were identified (Matlab function *findpeaks*). The frequency at which the most prominent of these peaks occurred was logged for every trial. These peak frequency values were then separated into load conditions and averaged across trials. This value was obtained for each participant for 1-back and 2-back trials. Differences in theta peak frequency between the two load conditions were compared via a paired-samples t-test. To obviate the possibility that any difference in these values reflects a shift in the slope of the 1/f component of the EEG signal,[48] the IRASA method (Irregular Resampling Auto-Spectral Analysis[49]) was employed to remove the 1/f component from the signal. Additionally, to ensure that this result was not a consequence of any volatility in single-trial power spectra, the analysis was also conducted on smoothed frequency spectra. The spectra were smoothed prior to peak detection via a sliding mean average using the Matlab function *smoothdata*. The degree of smoothing was varied between 2 and 5 elements (approximately 0.2-1.0 Hz window). For all of the preceding peak-based analyses, a trial was discarded if no peak was detected in that trial. Less than 1% of trials were discarded in all variations of this analysis (regardless of whether IRASA or smoothing was employed).

Classification

To decode object vs. scene representations during WM maintenance, multivariate pattern analyses (MVPA) was performed with the MVPA-light toolbox.[76] To reduce computational time of classification, data were resampled to 200 Hz. Prior to classification, data in the stimulus, response, and delay periods were smoothed with a running average (100 ms sliding window) and baseline-corrected to the preceding 200 ms. Trials were averaged within exemplar stimuli, as this has been shown to improve decoding performance.[77] In order to maintain a reasonable trial count this was only done by a factor of ~2. Trials of a given stimulus were randomly assigned to pairs and averaged, resulting in a single trial with presumed higher signal-to-noise ratio. Remainder trials (in the case of odd trials)

proceeded to classification unaveraged. For all classification analyses, linear discriminant analysis (LDA) was employed by taking the voltage values of the EEG channels as features at every time point. Classification was performed on all trials using a k-fold cross-validation procedure in which data were divided into 5 folds (4 training and 1 testing) in 5 iterations. This cross-validation procedure was repeated 5 times. The accuracy values across folds and repetitions were averaged to produce the final classifier performance. Decoding was conducted by training and testing classifiers on each time point of the task to generate a complete time by time temporal generalisation matrix. To examine whether the stimulus period generalised to the delay period, this time by time matrix was averaged across the training time dimension to the period when the stimulus was on the screen (0-750 ms), resulting in a time-series of average classifier accuracy across the testing time dimension (the time axis of the delay period). To determine which channels were most informative to correct classification, we implemented a searchlight approach in which, moving around the channel map of all 128 channels successively, an individual channel and its neighbours (radius = 0.10) were used to classify the data. The searchlight varied in size based on the number of neighbours, but on average the searchlight constituted 5.7 channels. The resultant accuracy for each of the searchlight centres was then tested against chance. Searchlight classification was performed by training and testing during the window in which significant above-chance decoding was observed including all channels (860-1275 ms into the delay period). As mentioned in the Results section, when assessing stimulus-to-delay generalisation and when decoding with only those channels that showed the coherence effect, accuracy values were again averaged across this previously defined temporal window of interest (860-1275 ms). The result of these analyses remained the same when accuracies were averaged or corrected (via cluster-correction) across the full delay period (See Table S1). Finally, we also confirmed stimulus decodability during the DMS task (See Figure S3).

Connectivity

To assess load-dependent changes in functional connectivity, cross-spectral densities were computed across the full time period via the Fieldtrip function *mtmconvol* using the same settings as in the time-frequency decomposition described previously. Pairwise channel coherence in the theta range (4-8 Hz) between channel Fz and every other channel was derived in the delay period of the 1-back task and the DMS task. Data from the DMS task were sub-sampled 10 times to accommodate the lower trial count in the 1-back condition.[78] These sub-sampled coherence maps were averaged before condition contrasts. Note also that, the impact of trial count on coherence precluded the use of the inter-block baseline periods as a comparator for 1-back trials, as there were only 24 inter-block baseline periods. Coherence values were compared statistically between 1-back and DMS conditions via cluster-based permutation tests in the time during the delay period where there was significant decoding above chance (860-1275ms).[79] As noted in the Results section, coherence values from the 1-back task were also averaged across the full 2.5 seconds of the delay period and compared to those from the DMS task.

To determine whether theta coherence was also greater in the 2-back task relative to the DMS task, coherence values were compared with cluster correction (as described above), again averaging across the full delay period. Significant clusters emerged when contrasting both the 1-back task and the 2-back task delay period with the DMS task, showing increased coherence over central posterior channels. In order to assess whether there was a frequency shift between 1-back and 2-back tasks, coherence values were averaged across channels which were members of both the 1-back and 2-back clusters (as depicted in Figure S4A). Coherence values were further averaged over the full time delay period. Finally, coherence values from the 1-back task were subtracted from the 2-back task to reveal at which frequency in the theta band coherence was significantly greater in the 2-back task than in the 1-back task. The resulting 2-back – 1-back difference in coherence was then subjected to a paired-samples t-test (See Figure S4B).

QUANTIFICATION AND STATISTICAL ANALYSIS

Subject exclusion criterion

Behavioural accuracy was calculated as the proportion of correct responses out of all trials. For outlier analysis, a composite score for each participant was computed by taking the mean accuracy on all three tasks. Outliers were defined as any value more than 1.5 inter-quartile ranges below the lower quartile or above the upper quartile across all participants. As mentioned in the "experimental model and subject details" section, this resulted in two participants being removed from subsequent analyses due to outlying low behavioural accuracy.

Inferential statistics

An alpha level of 0.05 was used as the threshold for statistical significance and tests were conducted as 2-tailed. To control for multiple comparisons across dimensions when assessing load-dependent power changes (channel/ frequency/time) or when assessing searchlight classification (channel/time), non-parametric cluster-based permutation testing was employed.[79] Briefly, this is achieved by first testing the spatio-spectro-temporal data via conventional statistics. Clusters are then formed where significant values are adjacent in sensor space, frequency and time. A specific metric of this cluster, e.g. the sum of its t-values, can be compared to a distribution of permuted cluster statistics to determine whether that cluster is statistically significant. This permutation testing was based on the maximum sum of a cluster's t-values, 500 permutations and at least three neighbouring channels constituting a cluster. To control for multiple comparisons otherwise, the Holm correction was employed.[80] Given the use of trial count-sensitive analyses and the low number of incorrect trials, all trials were analysed to maximise statistical power. Importantly, findings were not dependent on this error trial inclusion (see Figure S5). All statistical details are available in the body text of the Results section or in the relevant figure legend. Statistical testing was done via Matlab[1] and R (Version 3.4.3).[4]

Current Biology, Volume *32*

Supplemental Information

Fronto-medial theta coordinates

posterior maintenance of working memory content

Oliver Ratcliffe, Kimron Shapiro, and Bernhard P. Staresina

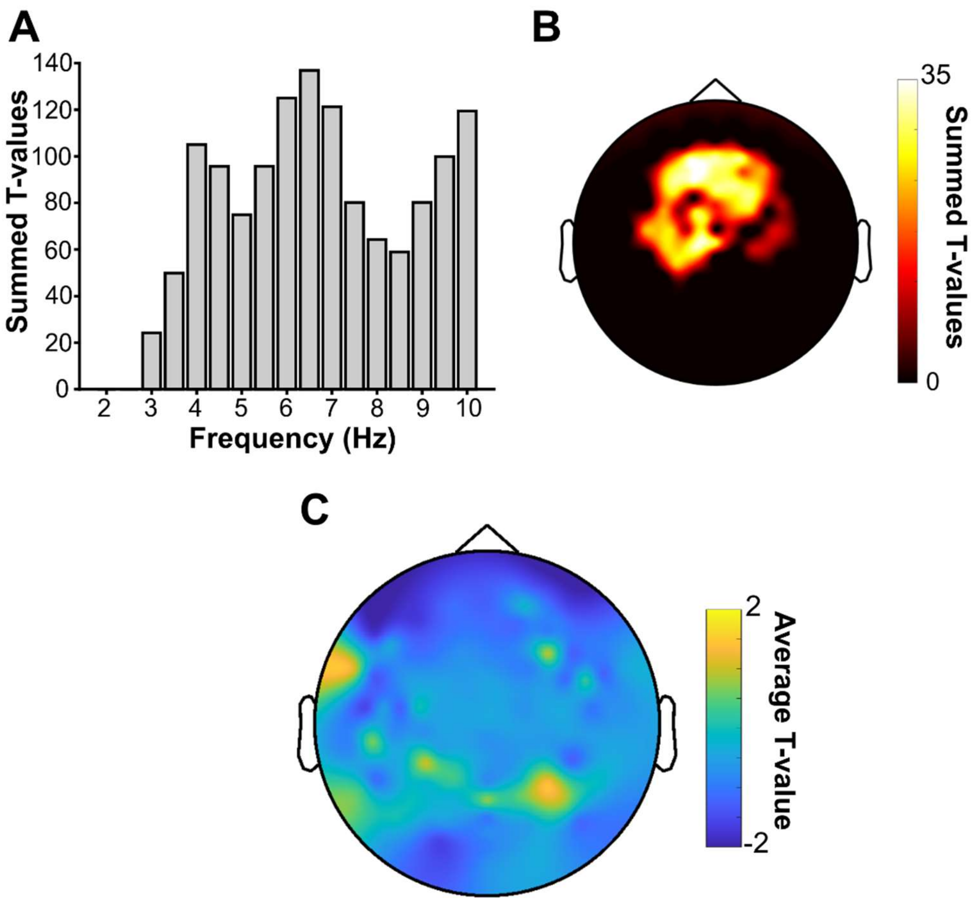

Figure S1. WM-induced fronto-medial theta (FMT) power in the delay period of the 1-back task (with an alternative baseline comparison) and in the delay period of the DMS task. Related to Figure 2. Cluster-corrected comparison of oscillatory power in the delay period of the 1-back task relative to an inter-block baseline period, revealing a significant increase in theta power (4-8 Hz; **A**, summed across significant channels) at fronto-medial channels (**B**, summed across significant frequencies from 4-8 Hz). **C.** Theta power in the delay period of the DMS task relative to the pre-stimulus baseline. No significant clusters emerged after correcting for multiple comparisons. The uncorrected topography (averaged over frequencies in the theta range) indicated that power was primarily increased over posterior channels, albeit without reaching statistical significance.

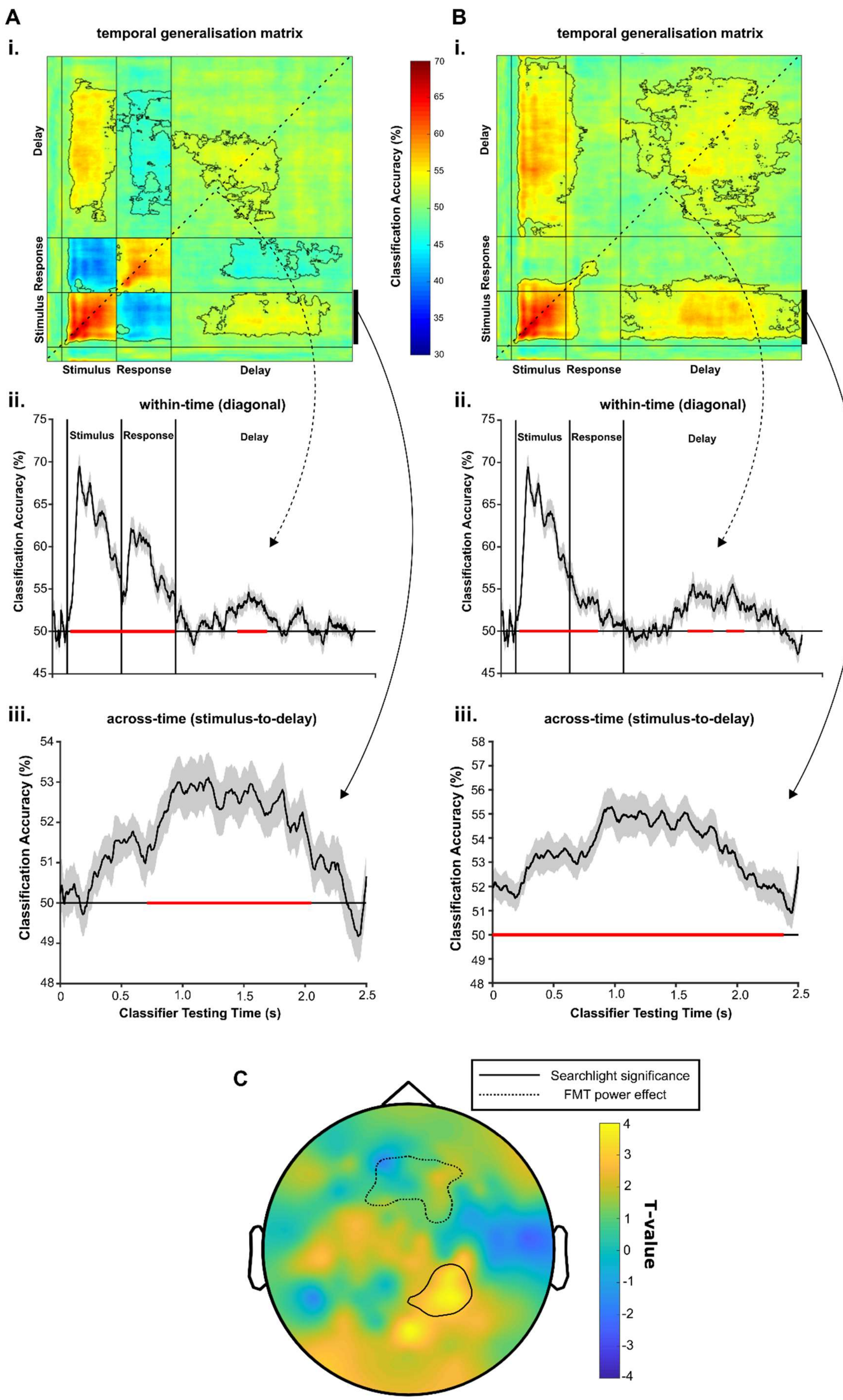
A
B
C
temporal generalisation matrix
i.
ii.
iii.
Delay
Stimulus Response
Stimulus Response
Classification Accuracy (%)
within-time (diagonal)
across-time (stimulus-to-delay)
Classifier Testing Time (s)
Searchlight significance
FMT power effect
T-value

Figure S2. Multivariate decoding across the full trial. Related to Figure 3. A.i. Classifiers trained and tested on every time point of each period of 1-back trials revealed generalisation between the stimulus and delay periods. Black outlines indicate cluster-corrected significance ($p < 0.05$). **A.ii.** Training and testing on the same time periods reveals significant decoding above chance in the stimulus, response, and delay periods. Note that these data reflect the diagonal of A.i. **A.iii.** Classifier accuracy was averaged across the training dimension during the period when the stimulus was on the screen (0-750 ms), revealing significant generalisation (as shown by above-chance accuracy) during the delay period. **B.** same as A., but conducting baseline correction using only the pre-stimulus period. **C.** Un-thresholded searchlight decoding topography. Topography of testing searchlight decoding accuracy against chance (cluster-corrected t-test); as in Figure 3B but plotting all channels (rather than only the significant cluster). Searchlight decoding was performed by using taking each channel and its immediate neighbours and decoding during the previously defined temporal window of interest (860-1275 ms). The value for each channel indicates the t-value (comparing the decoding performance relative to chance) when this channel was used as the centre of the decoding searchlight. The solid outline indicates cluster-corrected significance. To contrast, the dotted black line indicates channels showing the WM-induced FMT power difference (as in Figure 2B).

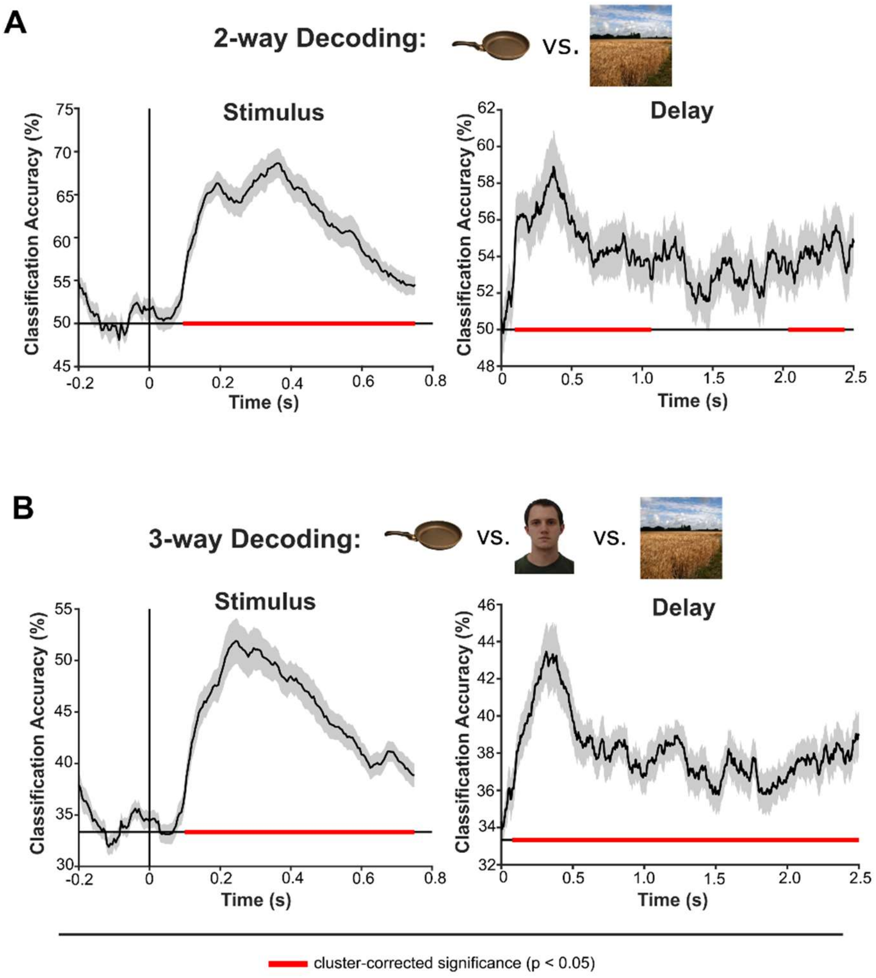

Figure S3. Decoding category of maintained content in the DMS task. Related to STAR★Methods. In order to test generalisability of 1-back maintenance decoding of WM representations to a similar task, we trained and tested classifiers on the stimulus and delay periods of the DMS task (main text Figure 1).

A. Binary decoding accuracy (classifying between object and scene trials) was significantly above chance during the stimulus and delay period intervals, as well as when averaging accuracy over the full time in each period [stimulus, $t_{(27)} = 11.46$, $p < 0.001$, Cohen's $d = 2.17$; delay, $t_{(27)} = 5.07$, $p < 0.001$, Cohen's $d = 0.96$]. **B.** Decoding between the three categories present in the DMS task (object/scene/face) was also above-chance during the stimulus and delay periods both when examining accuracy across-time and when averaging over the time dimension [$t_{(27)} = 10.87$, $p < 0.001$, Cohen's $d = 2.05$; delay, $t_{(27)} = 6.97$, $p < 0.001$, Cohen's $d = 1.32$].

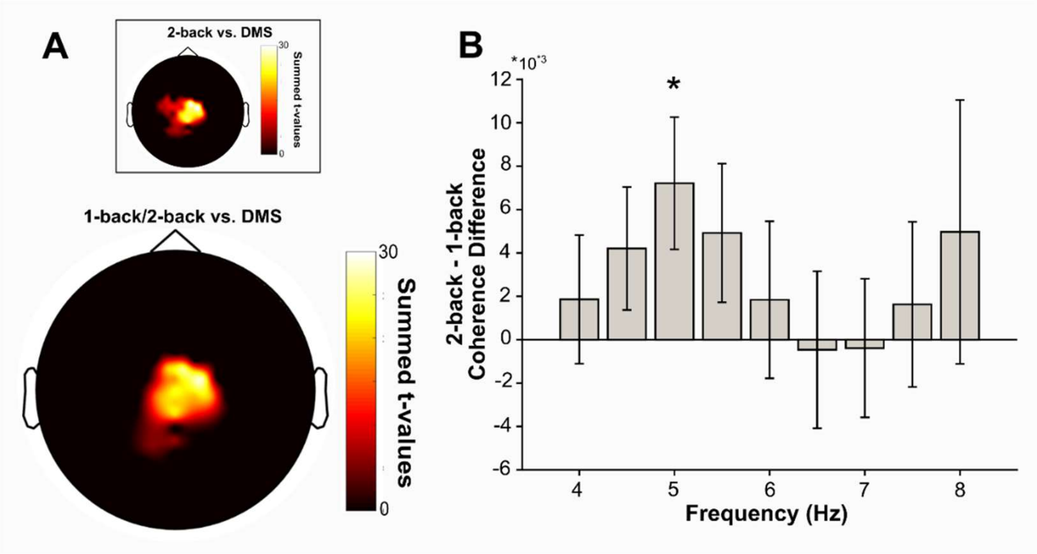

Figure S4. Theta coherence with increasing WM load. Related to STAR★Methods. A. Cluster-corrected comparison revealed an extended cluster showing increased coherence during the delay period of both the 2-back and 1-back tasks relative to the DMS task. T-values were summed across significant frequencies for each comparison and then averaged over the 1-back and 2-back tasks. **Inset.** Cluster showing significantly increased coherence in the delay period of the 2-back task relative to the DMS task. **B.** Averaging across the full delay period and significant channels, subtracting coherence values in the 1-back task from the 2-back task revealed a shift to the lower end of the theta band. This was evidenced by coherence at 5 Hz being significantly greater in the 2-back task relative to the 1-back task. Error bars indicate the standard error of the mean and the asterisk indicates statistical significance at alpha = 0.05.

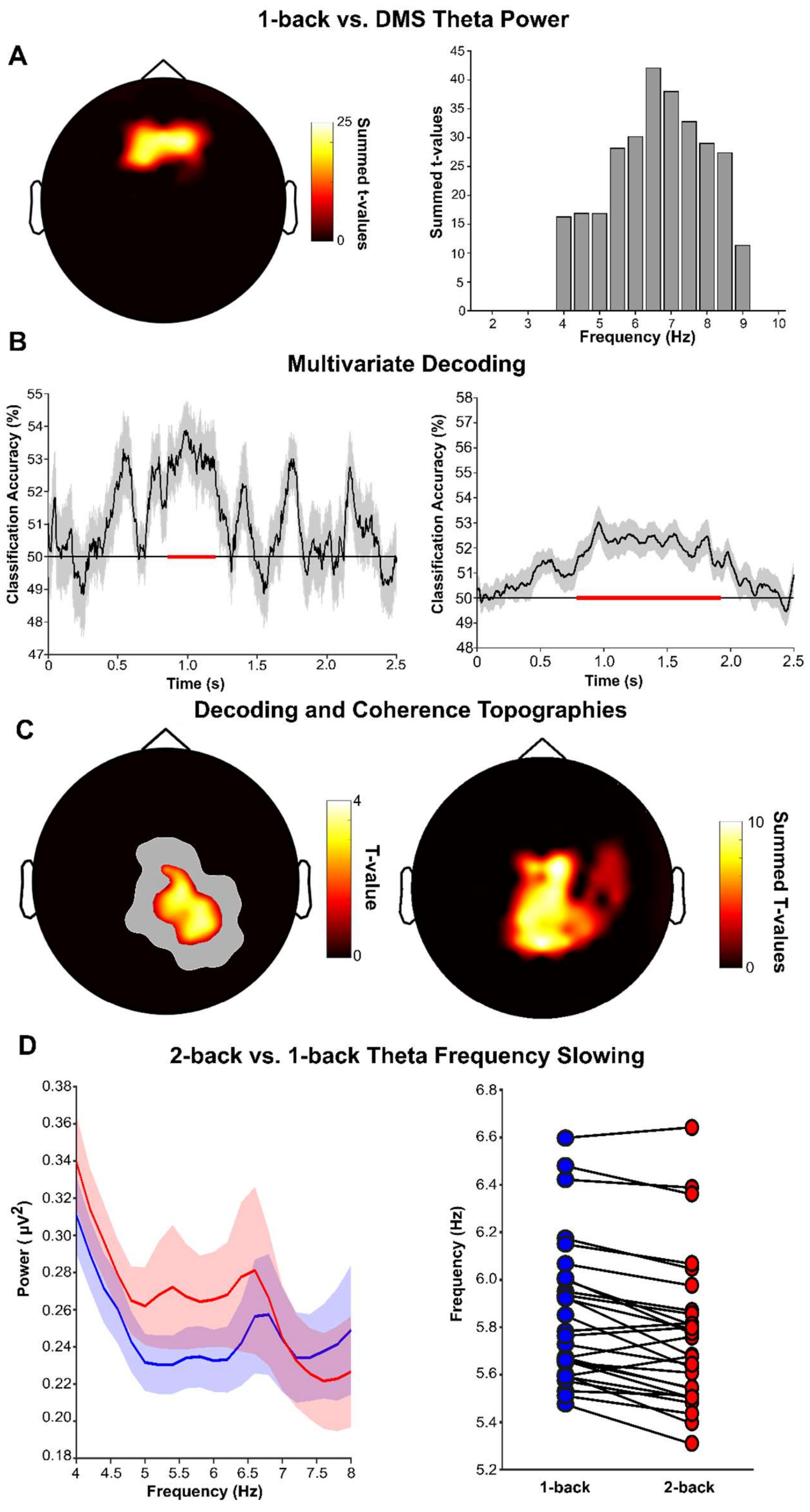

Figure S5. Analyses replicated using only correct trials. Related to STAR★Methods. A. FMT power effect comparing the delay period of the 1-back task to the same period during the DMS task. **B.** Accuracy of a classifier decoding the category of maintained content was significantly above chance during the delay period, both when training the classifier on that same period of the task **(left)** and when training on the stimulus presentation period of the task **(right)**. **C.** A decoding searchlight revealed that channels driving the significant decoding of stimulus representations showed substantial overlap with channels showing coherence between FMT channels in A. **D.** As when analysing all trials, the frequency of the FMT power effect significantly decreased in frequency (slowed) with the addition of a second to-be-remembered item during the delay period of correct trials

Comparison	t-value	p-value	Cohen's d	Cluster-corrected time (ms)
**Stimulus-to-delay generalisation**	4.40	<0.001*	0.83	710-2050
**Decoding with channels showing coherence with FMT activity**	2.46	0.02*	0.46	700-970

Table S1. Decoding across the full delay period. Related to STAR★Methods. Table displaying t-values, p values, and effect sizes averaging classifier accuracies (columns 2-4) and the time course of cluster-corrected periods of above-chance accuracy (column 5) across the full delay period (t-tests against chance). Asterisk indicates statistical significance ($p < 0.05$).
